# Amcinonide Exhibits Antitumor Effects in Radioresistant Colorectal Cancer Through the circRNF10/miR‐296‐5p/Snail Axis

**DOI:** 10.1002/advs.78502

**Published:** 2026-10-26

**Authors:** Hyun Jeong Seok, Dong Hyeon Lee, Junhye Kwon, Ui Sup Shin, In Hwa Bae

**Affiliations:** ^1^ Division of Radiation Biomedical Research Korea Institute of Radiological & Medical Sciences Seoul Republic of Korea; ^2^ Medical Sciences Substantiation Center Korea Cancer Center Hospital Korea Institute of Radiological & Medical Sciences Seoul Republic of Korea; ^3^ Department of Surgery Korea Cancer Center Hospital Korea Institute of Radiological & Medical Sciences Seoul Republic of Korea

**Keywords:** amcinonide, circRNF10, drug repurposing, miR‐296‐5p, radioresistant colorectal cancer, snail

## Abstract

Radiotherapy, commonly used in the treatment of colorectal cancer (CRC), can induce local recurrence and malignant progression in cases of intrinsic and acquired radioresistance; therefore, effective treatment strategies are required. In this study, we screened an FDA‐approved drug library and identified amcinonide (AMC), a topical corticosteroid, as a potential agent that inhibits radiation‐induced epithelial‐mesenchymal transition (EMT), cell migratory ability, and invasiveness in radioresistant colorectal cancer cells. Mechanistically, ionizing radiation‐induced circRNF10 suppressed miR‐296‐5p, thereby increasing Snail expression and promoting EMT and aggressive phenotypes in radioresistant CRC cells. We verified in vitro, in vivo, and in CRC patient‐derived organoids (PDOs) models that AMC suppresses the malignant phenotype by restoring miR‐296‐5p expression and reducing Snail expression. These findings identify the circRNF10/miR‐296‐5p/Snail axis as a key factor in CRC radioresistance and suggest that it may serve as a therapeutic target. Together, these results suggest that AMC is a promising drug for overcoming CRC radioresistance and that components of this axis may serve as potential diagnostic biomarkers. Overall, these findings highlight the therapeutic potential of targeting the ncRNA‐mediated EMT circuit to overcome radioresistant CRC.

## Graphical abstract

Amcinonide (AMC), the FDA‐approved corticosteroid, was identified as a repurposed agent for radiation‐resistant colorectal cancer. AMC suppresses radiation‐induced epithelial–mesenchymal transition, migration, and invasion via the circRNF10/miR‐296‐5p/Snail axis. It also inhibits exosome‐mediated M2 macrophage polarization and TGF‐β1 secretion, highlighting therapeutic and diagnostic biomarker potential. These findings suggest AMC contributes to overcoming radioresistant colorectal cancer by modulating the tumor and microenvironment.

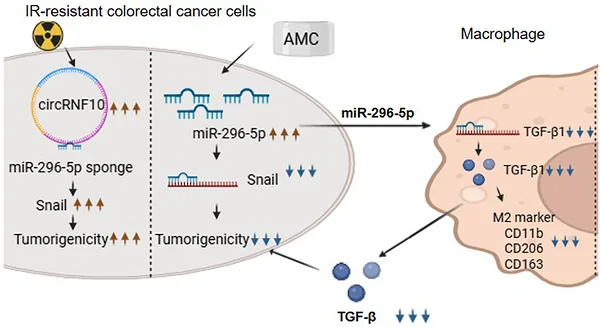

## 1 Introduction

Colorectal cancer (CRC) is the third leading cause of cancer‐related deaths worldwide. Radiotherapy is an important treatment option, particularly for stage II and III CRC [1], but the local recurrence rate in patients with colorectal cancer after treatment ranges from 15% to 65%. Thus, intrinsic and acquired radioresistance can promote malignant phenotypes such as accelerated EMT, invasiveness, and metastasis, severely limiting the effectiveness of CRC treatment [2, 3]. Therefore, the identification of effective candidate drugs to overcome radioresistance is urgently needed to improve the outcomes of CRC radiotherapy.

New drug development takes 12 to 15 years and costs between $1.5 and $2.5 billion, yet more than 90% of compounds fail in clinical trials, and fewer than 5% receive approval [4]. In contrast, drug repurposing can shorten the development period to 3 to 5 years and reduce costs by more than 85% by leveraging the established safety and pharmacokinetic properties of FDA‐approved drugs to identify new anticancer applications [4]. Representative examples include chlorambucil and busulfan, alkylating agents originally derived from mustard gas and repurposed for leukemia treatment [5], as well as thalidomide, a teratogenic drug later repurposed for multiple myeloma [6]. These examples highlight the potential of drug repurposing to identify new therapeutic uses. Accordingly, drug repurposing has become a widely used strategy for discovering new treatment indications, particularly in cancer therapy [7].

For drug repurposing, we focused on non‐coding RNAs (ncRNA) associated with radioresistance in CRC. Non‐coding RNAs, including miRNAs (microRNAs), lncRNAs (long non‐coding RNAs), and circRNAs (circular RNAs), regulate gene expression in tumor microenvironment (TME) constituent cells, such as cancer‐associated fibroblasts (CAFs), endothelial cells, and immune cells, and can also be delivered to these cells via tumor cell‐derived, thereby reprogramming the TME into a pro‐tumor and pro‐resistant state. For example, in CRC, the lnc‐TLCD2‐1/miR‐193a‐5p/YY1‐NF‐κB axis [8], the circ‐MFN2/miR‐574‐3p/IGF1R axis [9], and the exosome circ_IFT80/miR‐296‐5p/MSI1 axis [10] are representative ncRNA networks closely associated with the TME that regulate radioresistance as well as immune and inflammatory responses, invasion, and metastasis. However, the mechanism by which ncRNAs contribute to radioresistance in CRC remains incompletely understood.

This study identified the circRNF10/miR‐296‐5p/Snail axis as a key factor regulating radioresistance in CRC. circRNF10, whose expression is increased by ionizing radiation, relieves Snail inhibition by suppressing its target, miR‐296‐5p, thereby promoting EMT and malignant progression in radioresistant CRC cells. In particular, the study revealed that miR‐296‐5p contributes to inhibiting the malignancy mechanisms of radioresistant colorectal cancer by suppressing ionizing radiation‐induced tumorigenicity. Amcinonide (AMC), identified through drug repurposing, restored miR‐296‐5p expression, inhibited Snail‐driven EMT, and attenuated the aggressive behavior of radioresistant CRC in vitro, in vivo, and in PDOs models. These findings suggest that AMC may be a promising agent for overcoming CRC radioresistance and that factors of the circRNF10/miR‐296‐5p/Snail axis may serve as potential diagnostic biomarkers in CRC.

## 2 Results

### 2.1 AMC Inhibits the Tumorigenicity of Radioresistant Colorectal Cancer Cells by Downregulating Snail

In a previous study, we established radioresistant colorectal cancer cells (HCT116R and SW480R) and characterized them by analyzing cell viability, the expression of the DNA damage indicator γ‐H2AX protein, and colony formation ability according to radiation dose by applying fractionated radiation (5 Gy × 15 times, total 75 Gy) to the parental cells [11].

First, to discover drugs capable of overcoming radioresistant colorectal cancer, we performed a primary screening by treating the two established radioresistant colorectal cancer cell lines with a library of 774 FDA‐approved compounds at a concentration of 20 µm for 48 h and measuring cell proliferation. After selecting eight drugs that showed an inhibitory effect on the proliferation of radioresistant cells, we additionally analyzed changes in epithelial‐mesenchymal transition (EMT) marker abundance in the secondary screening (Figure S1A). Among these, B10 (amcinonide; AMC) was selected due to its pronounced suppression of EMT marker levels (Figure S1B). AMC is a topical corticosteroid currently used to treat inflammatory skin conditions such as eczema and psoriasis [12]. To further assess its effects, parental and radioresistant CRC cells were treated with various concentrations (20, 40, 60, 80, 100, 150, 200, 300, 350, and 400 µm) of AMC, and cell viability was measured (Figure S1C). At this time, the IC_50_ values of AMC were approximately 178.6 µm in HCT116 cells, 309.8 µm in HCT116R cells, 185.2 µm in SW480 cells, and 296.4 µm in SW480R cells, respectively. AMC reduced cell viability in a dose‐dependent manner not only in parental cells but also in radioresistant cells that are generally resistant to conventional therapies. These results suggest that AMC may play a significant role in inhibiting tumorigenicity in radioresistant CRC cells.

To investigate the underlying mechanism of AMC in more detail, follow‐up experiments were conducted using 80 µm, a concentration with minimal toxicity. This concentration further confirmed that no toxicity was observed in cell viability data in human intestinal epithelial cell‐6 (HIEC‐6) and human umbilical vein endothelial cells (HUVEC) (Figure S1D). In addition, flow cytometry analysis after PI staining showed that cytotoxicity did not significantly increase in cells treated with 80 µm AMC compared to the control group. Meanwhile, cells treated with 150 µm AMC, which served as the positive control, exhibited high cytotoxicity (Figure S1E). Collectively, these results demonstrate that AMC selectively inhibits radioresistant CRC cells without inducing non‐specific cytotoxicity, proving that 80 µm is an optimal and safe concentration for further mechanistic studies.

To evaluate the effects of AMC on tumorigenicity in radioresistant CRC cells, we assessed EMT markers, cell migration, and invasion, which are hallmarks of malignant phenotypes. Radioresistant cells exhibited significantly elevated abundance of EMT markers, including Snail, Zeb1, Twist, Slug, and Vimentin (Figure 1A), as well as increased migration (Figure 1B), and invasion (Figure 1C), compared to parental cells. Notably, AMC treatment markedly suppressed these changes. These results suggest that AMC has therapeutic potential for inhibiting malignant phenotypes in radioresistant CRC.

**FIGURE 1:**
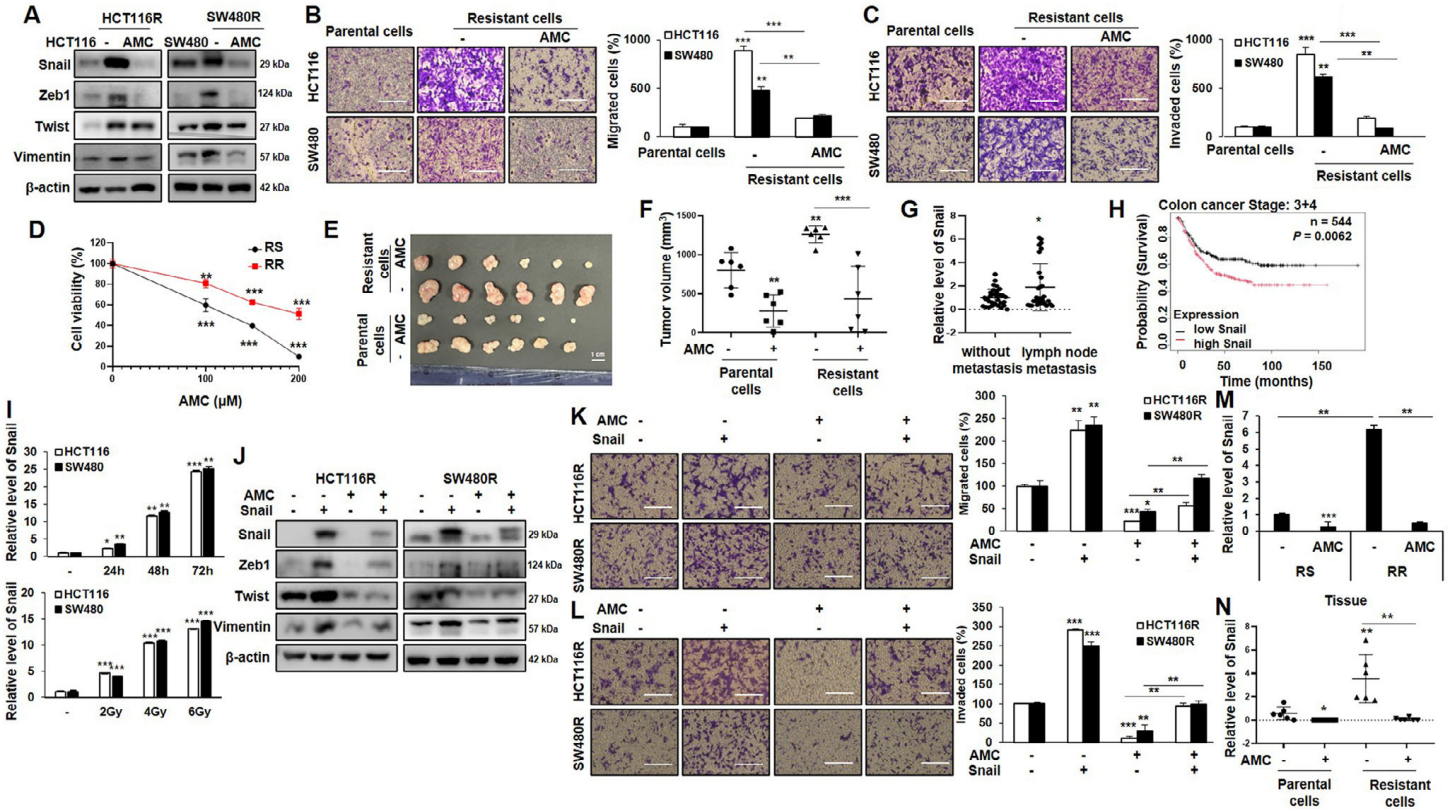
AMC reduces the tumorigenic capacity of radioresistant CRC cells by suppressing Snail levels. (A–C) HCT116 and SW480‐derived radioresistant cells were treated with AMC (80 µm), and EMT marker protein levels, cell migratory ability (B), and invasiveness (C) were assessed by western blotting, transwell migration (B, scale bar 100 µm), and Matrigel invasion assay (C, scale bar 100 µm). β‐Actin was used as a loading control. (D) PDOs from RS or RR CRC patients were treated with AMC at concentrations of 10, 25, 50, 100, 150, and 200 µm, and cell viability was evaluated using the WST‐8 assay. (E, F) Parental CRC cells (HCT116) and radioresistant cells (HCT116R) were injected subcutaneously into the right thigh of Balb/c nude mice. AMC (7 mg/kg) was administered intraperitoneally 14 times over 3 weeks, starting 1 week after injection. On day 28, mice were sacrificed, and tumors were excised. Tumor sizes (E) and volumes (F) were compared among groups (parental or radioresistant cells: NC, n = 6; AMC group, n = 6) (scale bar 1 cm). (G) Snail mRNA level in the plasma of CRC patients with or without lymph node metastasis was confirmed by qRT‐PCR. (no metastasis, n = 30; with metastasis, n = 32) (H) The correlation between relapse‐free survival and Snail expression in stage 3 and 4 CRC patients was shown using a Kaplan‐Meier plotter (https://kmplot.com). (I) Snail protein levels in HCT116 and SW480 cells were measured using qRT‐PCR according to the results at 24, 48, and 72 h after 5 Gy treatment (top) or 48 h after 2, 4, and 6 Gy exposure (bottom), respectively. (J–L) HCT116R and SW480R cells overexpressing Snail were treated with AMC (80 µm), and EMT marker expression (J), migration (K), and invasion (L) were determined by western blotting, transwell migration (K, scale bar 100 µm), and Matrigel invasion assays (L, scale bar 100 µm), respectively. (M) Snail mRNA level was measured by qRT‐PCR in PDOs derived from RS and RR CRC patients treated with AMC (80 µm). (N) Snail level was measured by qRT‐PCR in tumor tissues of mice injected with HCT116 or HCT116R cells, with or without AMC treatment. qRT‐PCR values were normalized to GAPDH. The data are presented as the mean ± SD for triplicate experiments. ^*^ *p* < 0.05, ^**^ *p* < 0.01, and ^***^ *p* < 0.001 were analyzed using Student's t‐Test for D, G, I, and M or One‐Way ANOVA for B, C, F, K, L, and N, respectively.

PDOs exhibit a high concordance rate of over 80% with clinical outcomes, making them a robust predictive model for personalized medicine [13]. To evaluate the clinical applicability of AMC, we assessed its effects on cell growth in radiosensitive (RS) and radioresistant (RR) CRC PDOs. Consistent with the results observed in intracellular experiments (Figure S1C), AMC significantly reduced cell viability in RR organoids as well as RS organoids, thereby supporting the potential of AMC as an anticancer agent for radioresistant CRC (Figure 1D).

To evaluate the in vivo effects of AMC, HCT116 and HCT116R cells were subcutaneously injected into mice, followed by intraperitoneal administration of AMC (7 mg/kg) 14 times over 3 weeks. There was no change in the body weight of the mice during the AMC administration period (Figure S1F). Tumors were harvested 28 days after injection, and tumor size (Figure 1E) and volume (Figure 1F) were measured. Tumors derived from radioresistant HCT116R cells exhibited the greatest growth, whereas AMC treatment significantly suppressed tumor growth.

At the cellular level, AMC significantly reduced Snail levels (Figure 1A). To explore the clinical relevance of Snail as a potential circulating biomarker, we analyzed plasma Snail levels in patients who underwent radiotherapy. As a result, Snail levels were significantly higher in patients who developed lymph node recurrence than in those who did not (Figure 1G). In addition, among patients with advanced‐stage disease and lymph node metastasis, elevated Snail levels were associated with a poorer prognosis (Figure 1H). Furthermore, Snail levels increased in a radiation time‐ and dose‐dependent manner (Figure 1I). Consequently, Snail, a key regulator of tumor malignancy, was identified as the most significantly downregulated target following AMC treatment and was selected for mechanistic analysis. We confirmed that AMC suppressed EMT marker abundance (Figure 1J), cell motility (Figure 1K), and invasiveness (Figure 1L), which were enhanced by Snail overexpression in radioresistant CRC cells. These results suggest that the tumor‐suppressive effects of AMC are mediated through the inhibition of Snail. Furthermore, Snail levels were significantly elevated in RR‐PDOs compared to the RS controls and were effectively reduced by AMC treatment (Figure 1M). Consistent with the in vitro results, Snail levels in mouse tumor tissues were highest in tumors derived from radioresistant CRC cells and were markedly suppressed by AMC administration (Figure 1N). Collectively, these results demonstrate that elevated Snail levels are associated with poor prognosis following radiotherapy, and that targeting Snail with AMC may represent a potential therapeutic strategy to overcome radioresistance‐associated tumor malignancy.

To investigate the effect of AMC on radiosensitivity in radioresistant CRC cells, we examined cell viability and tumorigenicity after radiation and/or AMC combination treatment. In HCT116R and SW480R cells, combined treatment with radiation and AMC significantly reduced cell viability in a dose‐dependent manner compared with radiation alone (Figure S2A). Furthermore, the combination treatment markedly reduced EMT markers (Figure S2B), cell motility (Figure S2C), and invasiveness (Figure S2D). Consistent with these in vitro findings, combined treatment with radiation and AMC significantly reduced tumor volume in a subcutaneous xenograft model compared with radiation alone (Figure S2E,F). These results demonstrate that AMC inhibits tumorigenesis by enhancing radiosensitivity in radioresistant CRC cells.

To investigate the effects of 5‐fluorouracil (5‐FU), a standard treatment for CRC, on radioresistant CRC cells, parental and radioresistant CRC cells were treated with 5‐FU at various concentrations (5, 10, 20, 30, 40, 50, 60, 80, 100, 150, 200, 250, 300, and 350 µm), and cell viability was measured (Figure S3A). At this time, the IC_50_ values of 5‐FU were approximately 43.6 µm in HCT116, 83 µm in HCT116R, 42.6 µm in SW480, and 83 µm in SW480R cells, respectively. 5‐FU significantly increased the IC_50_ values ​​of radioresistant CRC cells compared to parental cells. To compare the antitumor effects of 5‐FU and AMC in two radioresistant CRC cell lines, cell motility (Figure S3B) and invasiveness (Figure S3C) were further investigated. In particular, AMC suppressed the malignant phenotype more effectively than 5‐FU in radioresistant CRC cells. These results suggest that AMC has the potential to address the resistance of radioresistant CRC to existing therapies.

For further verification, 5‐FU‐resistant cells (5FUR) were constructed by treating HCT116 and SW480 cells to 16 and 14 cycles of 5‐FU treatment at concentrations of 1, 3, 5, 10, 20, and 30 µm, respectively. Compared with parental cells, the 5‐FU‐resistant CRC cells exhibited increased colony‐forming capacity (Figure S3D) and cell viability (Figure S3E), confirming the establishment of 5‐FU resistance. The IC_50_ value of 5‐FU was measured to be approximately 151.3 µm for HCT116 5FUR cells and approximately 251.8 µm for SW480 5FUR cells. Since the IC_50_ values of 5FUR cells were higher compared to their respective parental cell lines, HCT116 (IC_50_, 43.6 µm) and SW480 (IC_50_, 42.6 µm), it was indicated that resistance to 5‐FU was significantly increased.

To confirm the anticancer effect of AMC in 5‐FU‐resistant cells, cell viability and tumorigenic phenotypes were assessed. As a result, the IC_50_ values of AMC were measured to be approximately 274.1 µm in HCT116 5FUR cells and 324.5 µm in SW480 5FUR cells. AMC decreased cell viability in both parental and 5‐FU‐resistant cells (Figure S3F). In both 5‐FU‐resistant cell lines, AMC treatment demonstrated significant antitumor activity, effectively reducing cell motility (Figure S3G) and invasiveness (Figure S3H) compared with 5‐FU. These findings suggest that AMC is a promising antitumor drug candidate capable of overcoming resistance to existing therapies in CRC.

### 2.2 AMC‐Induced miR‐296‐5p Inhibits the Tumorigenic Phenotype of Radioresistant CRC by Targeting Snail

To elucidate the mechanism underlying AMC‐mediated suppression of radioresistance, we focused on miRNAs. miRNAs have been extensively studied as potential therapeutic agents and diagnostic markers [14]. Using a miRNA target prediction tool, we screened for miRNAs targeting Snail, a known radioresistance factor, and identified four candidate miRNAs: miR‐5585‐3p, miR‐499a, miR‐296‐5p, and miR‐199a‐5p (Figure 2A). In subsequent validation experiments, miR‐296‐5p and miR‐199a‐5p exhibited the most pronounced inhibitory effects at the Snail level (Figure 2B). As a result of comparing the levels of the two miRNAs after AMC treatment, miR‐296‐5p was significantly more upregulated (Figure 2C). Since miR‐296‐5p also demonstrated a higher ability to inhibit cell motility and invasiveness in radioresistant CRC cells than miR‐199a‐5p, this factor was selected (Figure S4). Consistent with these results, miR‐296‐5p overexpression significantly reduced Snail protein levels in HCT116R and SW480R cells (Figure 2D). To confirm direct binding of miR‐296‐5p to Snail, we constructed wild‐type (WT) and mutant‐type (MUT) 3'UTR luciferase reporter constructs of Snail (Figure 2E). A luciferase assay demonstrated that miR‐296‐5p significantly reduced luciferase activity in the WT construct but not in the MUT construct (Figure 2F). These results suggest that miR‐296‐5p directly binds to the 3'UTR of Snail to suppress its abundance.

**FIGURE 2:**
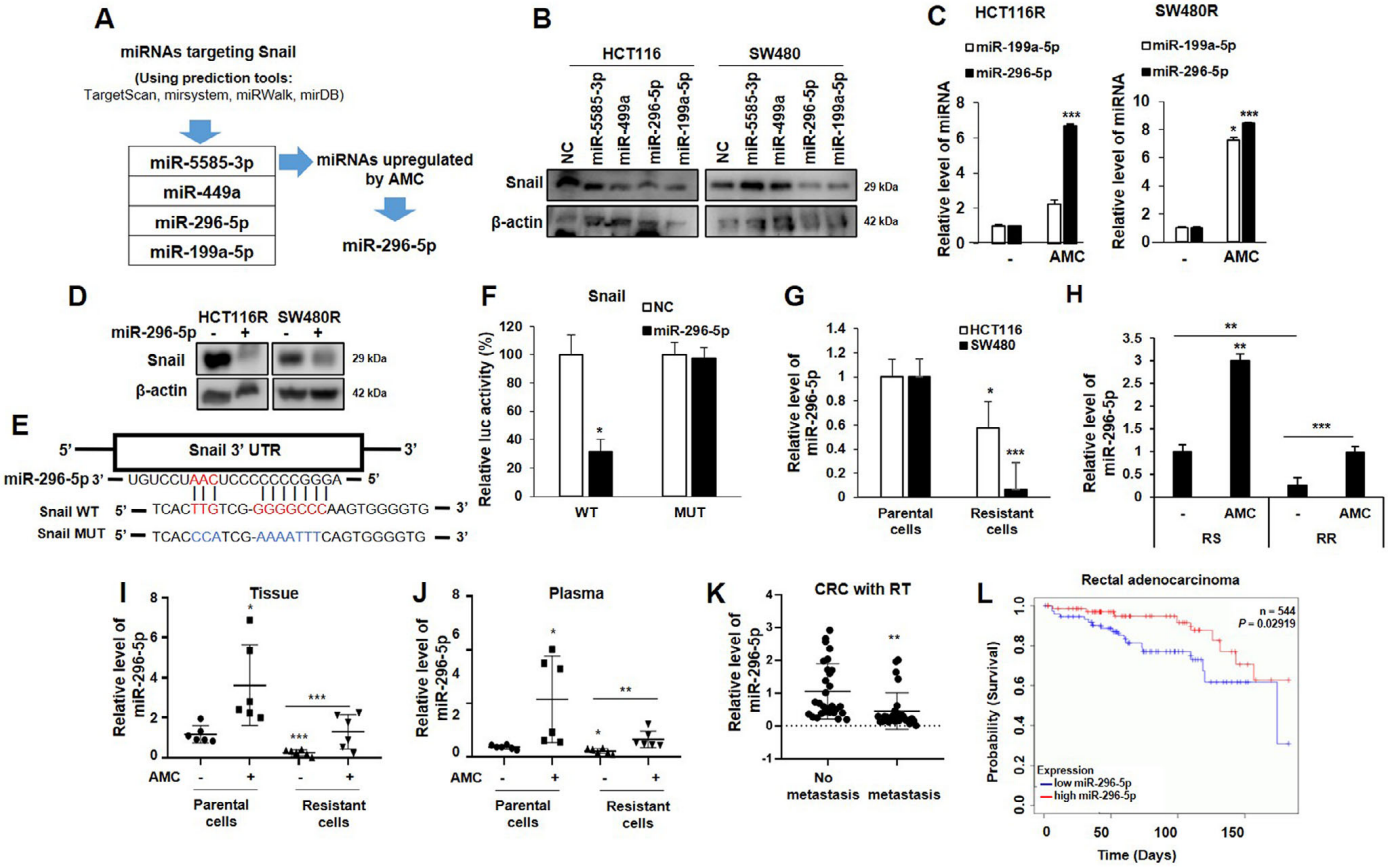
miR‐296‐5p, which directly targets Snail, is upregulated by AMC. (A) After candidate miRNAs targeting Snail were screened using miRNA target prediction tools (TargetScan, mirsystem, miRWalk, and mirDB), miR‐296‐5p, which was upregulated by AMC, was selected. (B) Four candidate miRNA mimics targeting Snail (miR‐5585‐3p, miR‐449a, miR‐296‐5p, and miR‐199a‐5p) were transfected into HCT116 and SW480 cells, and Snail protein expression levels were compared by western blotting. (C) After treatment of HCT116R and SW480R cells with AMC (80 µm), the levels of candidate miRNAs were assessed by qRT‐PCR. qRT‐PCR values were normalized to U6. (D) After transfecting HCT116R and SW480R cells with the selected miR‐296‐5p mimic, Snail protein levels were evaluated by western blot analysis. β‐Actin was used as a loading control. (E) The putative miR‐296‐5p binding site within the 3'UTR of Snail was synthesized as WT and MUT luciferase reporter constructs. (F) HCT116 cells were co‐transfected with the luciferase reporter constructs and a miR‐296‐5p mimic, and luciferase activity was measured 48 h. Firefly luciferase activity in each sample was normalized to Renilla luciferase activity. (G) miR‐296‐5p expression was compared by qRT‐PCR in parental cells (HCT116 and SW480) and radioresistant CRC cells (HCT116R and SW480R). (H) miR‐296‐5p expression in PDOs from RR‐ and RS‐CRC patients was analyzed by qRT‐PCR. (I‐J) qRT‐PCR was used to determine miR‐296‐5p expression in tumor tissues (I) and plasma (J) of mice subcutaneously injected with HCT116 and HCT116R cells, with or without AMC treatment. (K) miR‐296‐5p expression in plasma from CRC patients who received radiotherapy was analyzed by qRT‐PCR according to the presence or absence of metastasis (no metastasis, n = 29; lymph node metastasis, n = 29). (L) The prognostic significance of hsa‐miR‐296‐5p expression in patients with rectal adenocarcinoma (READ) was evaluated using OncoLnc (http://www.oncolnc.org). The data are presented as the mean ± SD for triplicate experiments. ^*^ *p* < 0.05, ^**^ *p* < 0.01, and ^***^ *p* < 0.001 were based on Student's t‐Test for C, F, G, H, and K or One‐Way ANOVA for I and J, respectively.

MiRNAs are known to exhibit disease‐specific profiles and have the potential to be used as diagnostic markers for various diseases [14]. Therefore, we investigated the association between miR‐296‐5p and radioresistant CRC. Compared to parental cells, miR‐296‐5p level was downregulated in radioresistant CRC cells (Figure 2G). Additionally, miR‐296‐5p level was lower in RR‐CRC patient‐derived organoids than in RS‐CRC patient‐derived organoids, and was increased upon AMC treatment (Figure 2H). Furthermore, miR‐296‐5p level was reduced in both tumor tissue (Figure 2I) and plasma (Figure 2J) of mice injected with radioresistant CRC, and was elevated in the AMC‐treated group. To further validate the clinical relevance of this finding in CRC patients, we analyzed miR‐296‐5p levels in patients who received radiation therapy. miR‐296‐5p level was lower in patients who developed lymph node metastases compared to those without metastases after radiation therapy (Figure 2K). Analysis of prognostic data from patients with rectal adenocarcinoma (READ) using OncoLnc (http://www.oncolnc.org) revealed that higher abundance of hsa‐miR‐296‐5p is significantly associated with improved overall survival (OS) (Figure 2L). Taken together, these results demonstrate that miR‐296‐5p, which directly suppresses Snail and is upregulated by AMC, is downregulated in radioresistant CRC compared to parental cells.

To determine whether AMC‐induced miR‐296‐5p mediates the antitumor effect, cells were co‐treated with AMC and a miR‐296‐5p inhibitor (anti‐miR‐296‐5p), and tumorigenic phenotypes were assessed. AMC effectively suppressed the increases in EMT marker abundance (Figure 3A), cell motility (Figure 3B), and invasiveness (Figure 3C) induced by the miR‐296‐5p inhibitor. Furthermore, to elucidate the involvement of miR‐296‐5p and its target Snail in tumor suppression, we examined the effects of miR‐296‐5p on Snail‐overexpressing cells. miR‐296‐5p suppressed the abundance of EMT markers (Figure 3D), cell motility (Figure 3E), and invasiveness (Figure 3F) increased by Snail overexpression. Collectively, these results demonstrate that AMC‐induced miR‐296‐5p suppresses tumorigenicity by downregulating Snail levels.

**FIGURE 3:**
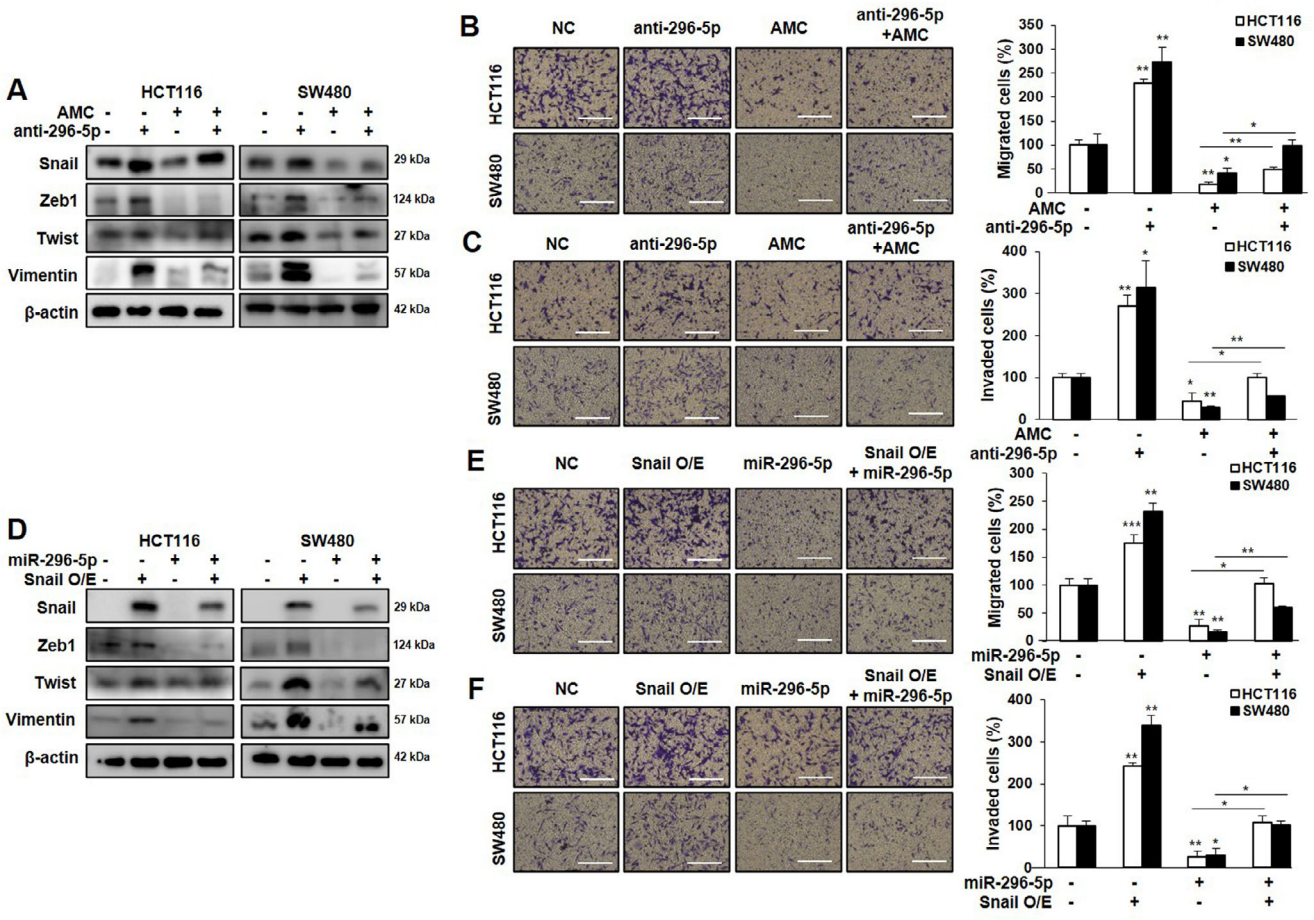
AMC‐induced miR‐296‐5p suppresses tumorigenic phenotypes by inhibiting Snail expression. (A–C) HCT116 and SW480 cells were transfected with miR‐296‐5p mimics and/or Snail overexpression vectors. Protein expression (A), cell migration (B), and invasion (C) were determined by western blotting, transwell migration (B, scale bar 100 µm), and Matrigel invasion assays (C, scale bar 100 µm). (D‐F) After transfection of HCT116 and SW480 cells with anti‐296‐5p and/or AMC, protein expression (D), cell migration (E), and invasiveness (F) were determined by western blotting, transwell migration (E, scale bar 100 µm), and Matrigel transwell invasion assay (F, scale bar 100 µm). The data are presented as the mean ± SD for triplicate experiments. ^*^ *p* < 0.05, ^**^ *p* < 0.01, and ^***^ *p* < 0.001 were analyzed using One‐Way ANOVA for B, C, E, and F.

### 2.3 circRNF10, A Sponge of miR‐296‐5p, Enhances Tumorigenicity by Showing High Expression Levels in Patients With Radioresistant CRC

CircRNAs are well known to function as miRNA sponges, and their disease‐specific abundance profiles have been extensively studied for their potential diagnostic markers [15]. To elucidate the mechanism underlying miR‐296‐5p suppression in radioresistant CRC cells, we identified relevant circRNAs. Initially, we selected eight candidate circRNAs predicted to target miR‐296‐5p using the miRNA‐circRNA prediction tools TargetScan and miRanda (Figure 4A). Using Gene Expression Omnibus (GEO) datasets, we identified circRNF10 (hsa_circ_0005043), which was upregulated in radioresistant CRC patients compared to radiosensitive CRC patients (Figure S5A). Additionally, an investigation into circRNA levels in parental (HCT116 and SW480) and radioresistant (HCT116R and SW480R) cells revealed that the amount of circRNF10 was increased in radioresistant CRC cells compared to parental CRC cells (Figure 4B and Figure S5B). Furthermore, we confirmed that the abundance of circRNF10 increased in a time‐ and dose‐dependent manner after irradiation (Figure 4C). circRNF10 is generated by back‐splicing of exons 11–14 from the RNF10 gene on chromosome 12 (Figure 4D). Overexpression of circRNF10 markedly reduced miR‐296‐5p abundance (Figure 4E). In summary, circRNF10, which is upregulated in radioresistant CRC, significantly inhibits miR‐296‐5p abundance by targeting it.

**FIGURE 4:**
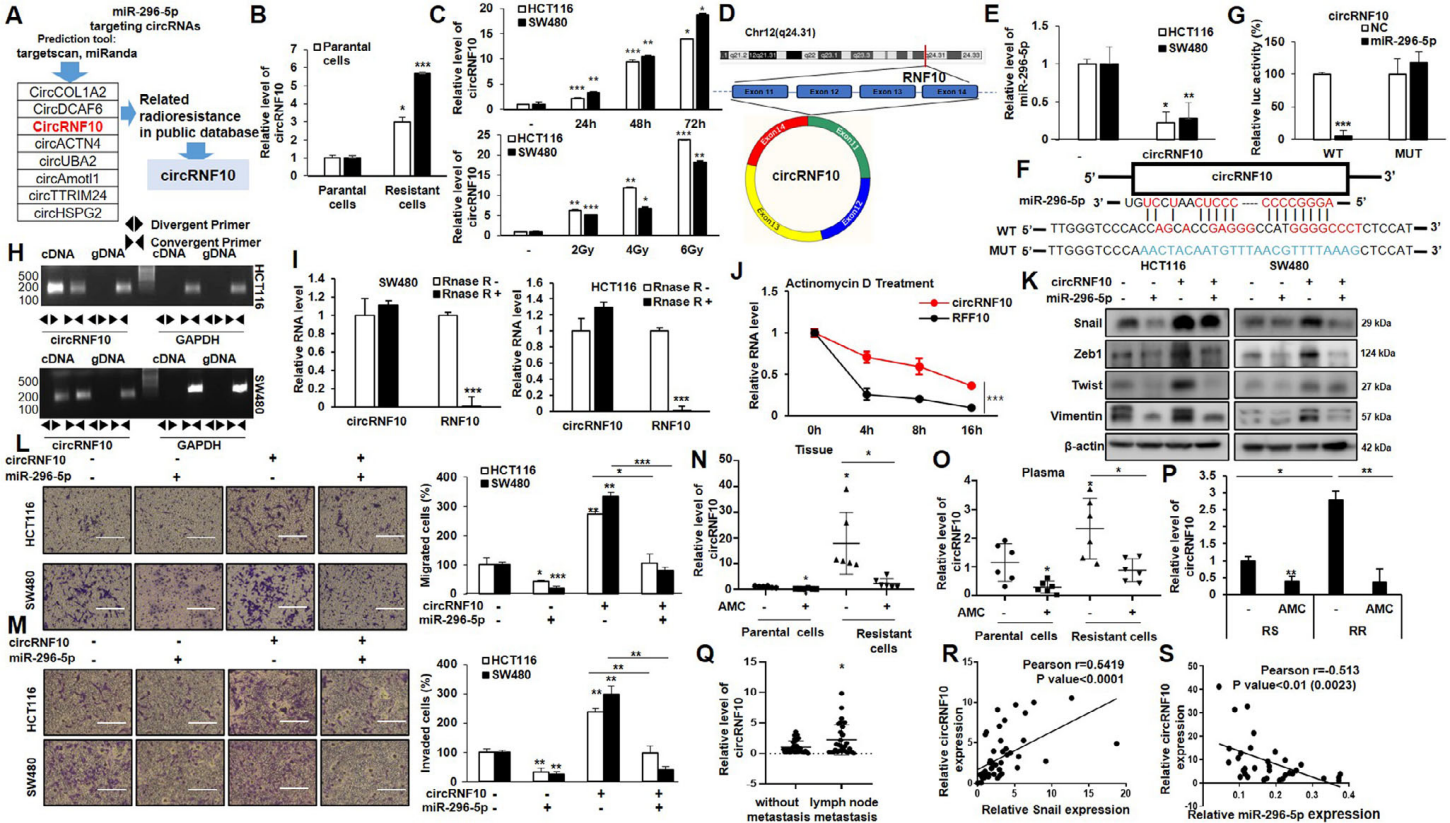
circRNF10 promotes CRC malignancy by suppressing miR‐296‐5p expression. (A) Among four circRNAs screened using TargetScan and miRanda, circRNF10 was selected based on its elevated expression in radioresistant CRC patients. (B) circRNF10 expression was compared in two parental and radioresistant CRC cells using qRT‐PCR. (C) circRNF10 levels in HCT116 and SW480 cells were measured using qRT‐PCR, respectively, according to results at 24, 48, and 72 h after 5 Gy treatment (top), or after 48 h after 2, 4, and 6 Gy treatment (bottom). (D) Schematic representation of the genomic location and splicing pattern of circRNF10. (E) After overexpressing circRNF10 in HCT116 and SW480 cells, the abundance of miR‐296‐5p was confirmed by qRT‐PCR. qRT‐PCR results were normalized to U6 and GAPDH, respectively. (F) The miR‐296‐5p binding site was cloned into circRNF10, forming WT and MUT constructs. (G) The WT and MUT constructs were co‐transfected with miR‐296‐5p into HCT116 cells, and luciferase activity was measured after 48 h. Firefly luciferase activity was normalized to Renilla luciferase activity of each sample. (H) circRNF10 expression was validated by PCR using divergent and convergent primers. (I) circRNF10 and linear RNF10 mRNA expression were analyzed by qRT‐PCR after RNase R treatment in HCT116 and SW480 cells. (J) circRNF10 and RNF10 mRNA expression were measured by qRT‐PCR at 4, 8, and 16 h after treatment with actinomycin D (10 µg/ml) in HCT116 and SW480 cells. (K‐M) HCT116 and SW480 cells were co‐transfected with circRNF10 and/or miR‐296‐5p, and EMT marker expression (K), cell motility (L), and invasion (M) were determined by western blotting, transwell migration (L, scale bar 100 µm), and Matrigel transwell invasion assay (M, scale bar 100 µm), respectively. (N, O) circRNF10 expression was determined by qRT‐PCR in tumor tissue (N) and plasma (O) of mice injected with parental and radioresistant CRC cells, with or without AMC treatment. (P) circRNF10 expression was assessed by qRT‐PCR in RS‐ and RR‐CRC PDOs, with or without AMC treatment. (Q) circRNF10 expression was evaluated by qRT‐PCR in plasma from CRC patients with or without lymph node metastasis (no metastasis, n = 32; lymph node metastasis, n = 30). (R–S) The correlation between circRNF10 and Snail levels (50 pairs, R) or circRNF10 and miR‐296‐5p levels (35 pairs, S) in plasma from CRC patients who received radiotherapy was analyzed using Spearman's rank correlation coefficients. The data are presented as the mean ± SD for triplicate experiments. ^*^ *p* < 0.05, ^**^ *p* < 0.01, and ^***^ *p* < 0.001 were based on Student's t‐Test for B, C, E, F, G, I, J, P, Q, R, and S or One‐Way ANOVA for L, M, N, and O, respectively.

To confirm direct binding of circRNF10 to miR‐296‐5p, we cloned WT and MUT luciferase reporter constructs containing the miR‐296‐5p binding site (Figure 4F). miR‐296‐5p overexpression reduced luciferase activity in the WT construct, but not in the MUT construct (Figure 4G). This indicates that circRNF10 directly binds miR‐296‐5p and inhibits its activity. To determine whether head‐to‐tail splicing of circRNF10 resulted from genomic rearrangements or PCR artifacts, we performed PCR using convergent and divergent primers. Divergent primers amplified circRNF10 only from cDNA, not gDNA, whereas convergent primers amplified the target from both cDNA and gDNA in both cell lines (Figure 4H). Furthermore, circRNF10 was resistant to RNase R exonuclease degradation, whereas RNF10 mRNA was susceptible (Figure 4I). To further confirm that circRNF10 has a stable ring structure, we treated HCT116 cells with actinomycin D and found that circRNF10 was substantially more stable than the RNF10 mRNA (Figure 4J). These findings confirm that circRNF10 directly interacts with miR‐296‐5p and exhibits canonical circRNA characteristics, including cDNA‐specific amplification, exonuclease resistance, and enhanced stability.

To determine whether circRNF10 acts upstream of miR‐296‐5p in mediating its tumor suppressive effects, we co‐transfected cells with a circRNF10 overexpression vector and a miR‐296‐5p mimic, and assessed tumorigenic phenotypes. Overexpression of miR‐296‐5p markedly reversed circRNF10‐induced EMT marker abundance (Figure 4K), cell motility (Figure 4L), and invasiveness (Figure 4M). In addition, to determine whether AMC suppresses malignancy through the downregulation of circRNF10, a resistance‐related factor, we co‐treated circRNF10 overexpression with AMC. As a result, AMC reduced circRNF10 level (Figure S6A) and reversed the circRNF10‐mediated elevation of EMT marker abundance (Figure S6B), cell motility (Figure S6C), and invasiveness (Figure S6D). Furthermore, we investigated the abundance of Snail, miR‐296‐5p, and circRNF10 following treatment with AMC and the GR antagonist RU‐486. As a result, the decrease in circRNF10 expression (Figure S7A), the increase in miR‐296‐5p expression (Figure S7B), and the decrease in Snail levels (Figure S7C) caused by AMC were effectively reversed by RU‐486 treatment. Taking these results together, AMC suggests a potential role for overcoming resistance‐related tumorigenicity by suppressing malignant characteristics through the circRNF10/miR‐296‐5p/Snail axis.

In vivo, circRNF10 level was markedly elevated in both tumor tissue (Figure 4N) and plasma (Figure 4O) of the radioresistant group compared to the radiosensitive group, and was reduced by AMC treatment. Similarly, in CRC PDOs, circRNF10 abundance was higher in the RR group than in the RS group and decreased upon AMC treatment (Figure 4P). Clinically, among patients receiving radiotherapy, circRNF10 abundance was significantly higher in patients with lymph node metastasis than in those without (Figure 4Q). Moreover, Snail showed a positive correlation with circRNF10 (Figure 4R) and a negative correlation with miR‐296‐5p in patients who received radiotherapy (Figure 4S). Overall, circRNF10 shows potential value as a predictive indicator of radiation‐resistant colorectal cancer along with the miR‐296‐5p/Snail axis.

AMC downregulates circRNF10 expression in vivo (Figure 4N) and in organoids (Figure 4P), but the detailed mechanism has not yet been elucidated. Considering that AMC acts as an agonist for the human glucocorticoid receptor (GR), which functions as a transcription factor [16], we hypothesized that GR transcriptionally regulates circRNF10 expression by binding to the promoter region of circRNF10. Since circRNA is generated through the reverse splicing of precursor mRNA, we reasoned that GR could regulate circRNF10 expression by controlling the transcription of the host gene RNF10. To verify this hypothesis, we analyzed the 1.5 kb upstream promoter region of RNF10 using the transcription factor binding prediction tool AliBaba 2.1 and identified eight potential NR3C1 binding sites. NR3C1 (nuclear receptor subfamily 3 group C member 1) encodes GR and mediates its transcriptional activity [17]. To confirm the potential for GR to bind to the RNF10 promoter, NR3C1 binding was verified using ENCODE ChIP‐seq data (GEO: GSE170210) obtained from CRISPR‐induced NR3C1 knockout HepG2 cell line samples. As a result, it was confirmed that NR3C1 binds strongly to sites 5 and 6 within this RNF10 promoter region (Figure S8A). In order to determine whether AMC (GR) actually affects the RNF10 promoter, we constructed RNF10 promoter constructs containing site‐specific mutations at the predicted NR3C1 binding sites and performed luciferase reporter analysis. As a result, mutations at the predicted binding sites (Figure S8B). As a result, mutations at the predicted binding sites 5 and 6 significantly reduced the AMC‐dependent transcriptional activity of the RNF10 promoter compared to the corresponding wild‐type promoter structure. These results provide direct functional evidence that the predicted NR3C1 binding site is involved in the transcriptional regulation of RNF10.

### 2.4 AMC‐Induced miR‐296‐5p Translocated to Macrophages, Where It Inhibiting M2 Polarization and TGF‐β1 Secretion

The tumor microenvironment significantly influences radiotherapy efficacy through complex interactions among cancer cells, surrounding stromal cells, immune cells, blood vessels, and hypoxic regions [18]. Therefore, this is a crucial aspect of research on radioresistance. Using the TISCH2 database, we identified tumor microenvironment cells associated with Snail, a key factor in the anticancer activity of AMC and a core mediator of radioresistance. Single‐cell RNA sequencing analysis (CRC_GSE139555 dataset—Pre‐treatment specimen data from 14 CRC patients, including tumors, normal adjacent tissues, and peripheral blood) within the TISCH2 revealed that Snail abundance correlated more strongly with immune cells than stromal cells (Figure S9), particularly with macrophages (Figure 5A–C). Previous studies have shown that Snail‐overexpressing cancer cells recruit macrophages via CXCL1 and CXCL2 secretion [19]. These macrophages adopt an M2‐like phenotype, secrete TGF‐β1, and form a positive feedback loop that sustains high Snail abundance in tumor cells [19].

**FIGURE 5:**
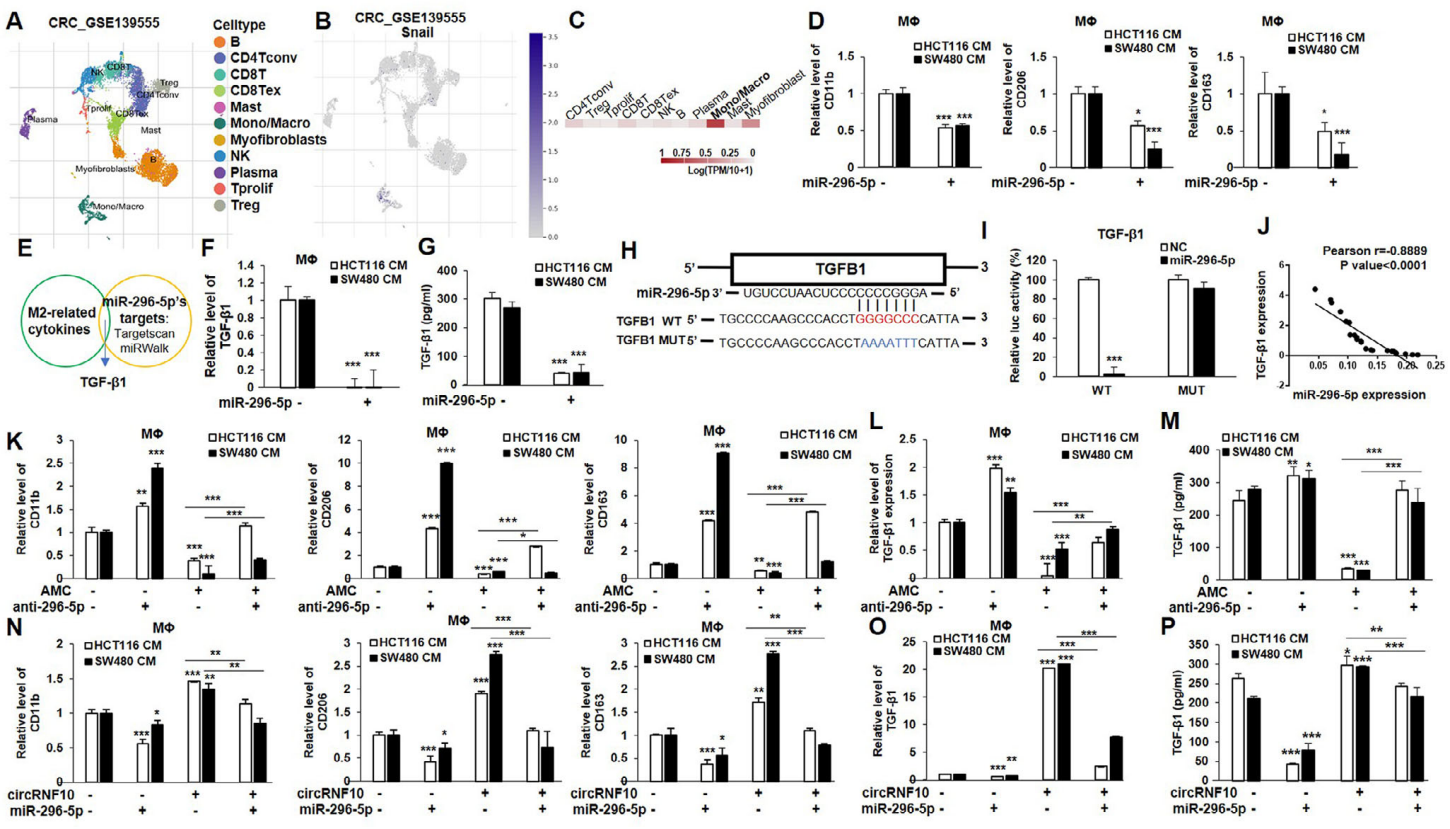
AMC‐induced miR‐296‐5p suppresses M2 polarization and TGF‐β1 secretion in macrophages. (A–C) Immune cell populations associated with Snail abundance were identified through single‐cell RNA sequencing analysis of a CRC dataset (CRC_GSE139555). (A–C) The distribution of various immune cell subsets (A) and the expression pattern of Snail (B) are shown in the single‐cell RNA sequencing analysis. (C) The heatmap displays the levels of Snail expression across different immune cell types identified by single‐cell RNA sequencing analysis. (D) After treating THP‐1‐derived macrophages with CM obtained by overexpressing miR‐296‐5p in HCT116 and SW480 cells, mRNA levels of M2 markers (CD11b, CD206, and CD163) were measured by qRT‐PCR. qRT‐PCR results were normalized to GAPDH. (E) The Venn diagram illustrates the common overlap between predicted miR‐296‐5p target genes and TGF‐β1, a cytokine secreted by M2‐polarized macrophages, indicating that TGF‐β1 is a candidate target of AMC‐induced miR‐296‐5p. (F, G) The mRNA levels (F) and secretion of TGF‐β1 (G) in THP‐1‐derived macrophages treated with CM of HCT116 and SW480 cells overexpressing miR‐296‐5p were analyzed by qRT‐PCR and ELISA, respectively. (H) WT or MUT reporter constructs containing the miR‐296‐5p binding site within the TGF‐β1 3′UTR were synthesized. (I) HCT116 cells were co‐transfected with a miR‐296‐5p mimic and either the WT or MUT constructs, and a dual‐luciferase reporter assay was performed. (J) The correlation between TGF‐β1 and miR‐296‐5p levels (23 pairs, S) in plasma from CRC patients who received radiotherapy was analyzed using Spearman rank correlation coefficients. (K–M) CM collected after treatment of HCT116 and SW480 cells with a miR‐296‐5p inhibitor and/or AMC was applied to macrophages, and the abundance of M2 markers (K), intracellular TGF‐β1 (L), and secreted TGF‐β1 (M) was analyzed by qRT‐PCR and ELISA, respectively. (N‐P) After macrophages were treated with CM from HCT116 and SW480 cells transfected with circRNF10 and/or miR‐296‐5p, the abundance of M2 marker (N), intracellular TGF‐β1 (O), and secreted TGF‐β1 (P) was assessed by qRT‐PCR and ELISA analysis. The data are presented as the mean ± SD for triplicate experiments. ^*^ *p* < 0.05, ^**^ *p* < 0.01, and ^***^ *p* < 0.001 were analyzed using Student's t‐Test for D, F, G, I, and J or One‐Way ANOVA for K, L, M, N, O, and P, respectively.

To investigate the role of miR‐296‐5p, a miRNA targeting Snail, in the interaction between CRC cells and macrophages, macrophages were treated with conditioned medium (CM) secreted by CRC cells overexpressing miR‐296‐5p, and the abundance of M2 polarization markers was analyzed. Macrophages exposed to miR‐296‐5p‐overexpressing CM exhibited reduced levels of M2 markers (CD11b, CD206, and CD163) compared to control CM‐treated macrophages (Figure 5D).

Since ncRNAs, such as miRNAs and circRNAs, are known to mediate intercellular signaling via exosomes [20], we hypothesized that exosomes would mediate the transfer of miR‐296‐5p from CRC cells to macrophages. To confirm this, we observed high expression of the exosome markers CD63, CD9, and CD81 in exosomes isolated from CM obtained after transfecting HCT116R and SW480R cells with Cy3‐labeled miR‐296‐5p mimics (Figure S10A), and qRT‐PCR analysis showed that the levels of miR‐296‐5p within exosomes were significantly increased (Figure S10B). Fluorescence microscopy confirmed Cy3 signaling not only in CRC cells (Figure S10C) but also in macrophages treated with CM of CRC cells (Figure S10D). In addition, we confirmed that circRNF10 levels increased in exosomes isolated from HCT116R and SW480R cells overexpressing circRNF10 (Figure S10E). These results suggest that miR‐296‐5p and circRNF10 were transferred from CRC cells to macrophages via exosomes.

Functionally, inhibiting exosome secretion with GW4869 significantly suppressed the ability of CM from miR‐296‐5p‐overexpressing cells to induce M2 macrophage polarization by reducing the expression of M2‐related markers (Figure S10F). Furthermore, even when macrophages were treated with Actinomycin D to block transcription prior to CM exposure, intracellular miR‐296‐5p levels remained elevated, suggesting that miR‐296‐5p is not newly synthesized in macrophages but is delivered from CRC cells via exosome‐mediated signaling (Figure S10G). These results demonstrate that miR‐296‐5p is selectively packaged into CRC cell‐derived exosomes and delivered to macrophages, thereby contributing to M2 macrophage polarization.

To elucidate the mechanism underlying miR‐296‐5p‐mediated suppression of M2 polarization, we screened TGF‐β1 using M2‐related cytokines [21] and TargetScan and miRWalk target prediction (Figure 5E). TGF‐β1 is a well‐established inducer of M2 polarization in macrophages [22] and is abundantly secreted by M2 macrophages [23, 24]. Both TGF‐β1 mRNA abundance (Figure 5F) and secretion (Figure 5G) were reduced in macrophages treated with CM from miR‐296‐5p‐overexpressing HCT116 and SW480 cells.

Additionally, to confirm TGF‐β1 as a direct target of miR‐296‐5p, WT and MUT constructs containing the miR‐296‐5p binding sites within the 3'UTR of TGF‐β1 mRNA were constructed (Figure 5H) and analyzed used in luciferase assays (Figure 5I). Overexpression of miR‐296‐5p reduced luciferase activity in the wild‐type (WT) construct of the TGF‐β1 3'UTR, but no significant change was observed in its mutant‐type (MUT) construct (Figure 5I). These findings indicate that miR‐296‐5p directly binds to the 3′UTR of TGF‐β1 and negatively regulates its abundance. Additionally, the clinical significance in radioresistant CRC is further supported by confirming a negative correlation between TGF‐β1 levels and miR‐296‐5p expression levels in the plasma of CRC patients who have undergone radiotherapy (Figure 5J).

To determine whether AMC, which induces miR‐296‐5p, is involved in M2 macrophage polarization and TGF‐β1 secretion, macrophages were treated with CM from HCT116 and SW480 cells transfected with a miR‐296‐5p inhibitor, with or without AMC treatment, and M2 marker expression was analyzed. CM derived from AMC‐treated CRC cells decreased the abundance of M2 markers (Figure 5K) and TGF‐β1 mRNA (Figure 5L), as well as TGF‐β1 secretion (Figure 5M) in macrophages. Notably, these inhibitory effects were effectively reversed by CM from CRC cells transfected with the miR‐296‐5p inhibitor.

Additionally, to investigate the role of circRNF10, which negatively correlates with miR‐296‐5p, in M2 macrophage polarization, macrophages were treated with CM from circRNF10‐overexpressing CRC cells, and M2 polarization markers were assessed. Macrophages exposed to CM from circRNF10‐overexpressing cells exhibited increased abundance of M2 markers (Figure 5N), elevated TGF‐β1 mRNA abundance (Figure 5O), and increased TGF‐β1 secretion (Figure 5P), which were attenuated by CM from cells overexpressing miR‐296‐5p. Collectively, these results suggest that AMC‐induced miR‐296‐5p suppresses M2 polarization and TGF‐β1 secretion in tumor‐associated macrophages within the colon cancer microenvironment.

### 2.5 AMC Suppresses CRC Malignancy by Inhibiting M2 Macrophage Polarization and TGF‐β1 Secretion

To determine the effect of AMC on TGF‐β1 abundance in macrophages, we co‐treated macrophages with AMC and recombinant TGF‐β1 (rTGF‐β1). AMC reduced rTGF‐β1‐induced intracellular TGF‐β1 (Figure 6A), TGF‐β1 secretion (Figure 6B), and M2 polarization markers (Figure 6C) in macrophages. Because macrophage‐secreted TGF‐β1 is known to promote cancer cell malignancy via a positive feedback loop [23, 24], we hypothesized that miR‐296‐5p transferred to macrophages would inhibit TGF‐β1 abundance and thereby inhibit cancer cell malignancy. To verify this, we treated CRC cells with macrophage CM containing miR‐296‐5p mimic and rTGF‐β1, and examined their malignant characteristics. The rTGF‐β1‐induced increase in M2 polarization markers in macrophages was suppressed by miR‐296‐5p overexpression (Figure 6D). Additionally, HCT116 and SW480 cells treated with macrophage CM stimulated with rTGF‐β1 exhibited increased abundance of EMT markers (Figure 6E), enhanced cell motility (Figure 6F), and invasiveness (Figure 6G). Furthermore, treatment with macrophage CM overexpressing miR‐296‐5p suppressed all tumorigenic traits.

**FIGURE 6:**
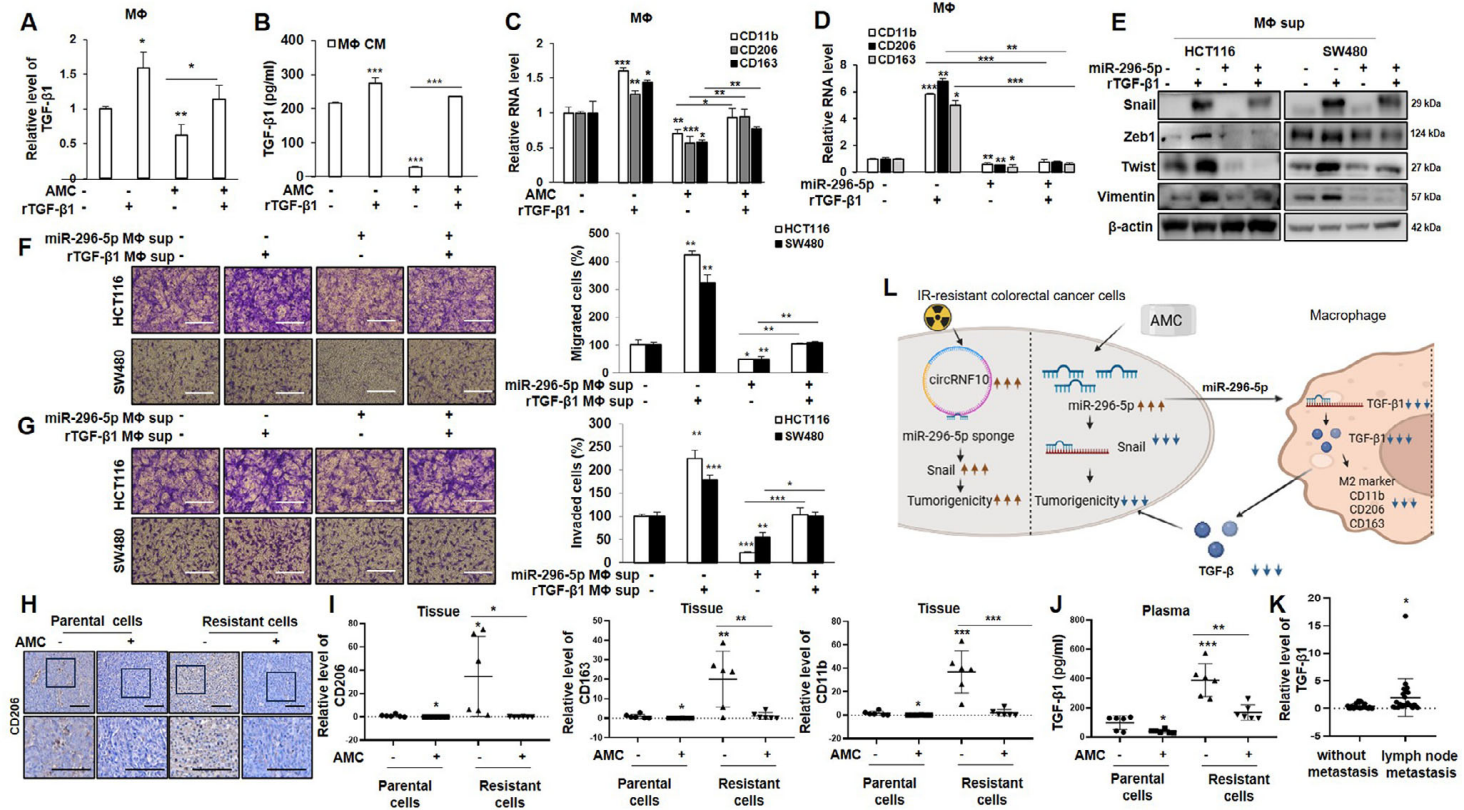
AMC suppresses CRC tumorigenicity by inhibiting M2 marker abundance and TGF‐β1 secretion in macrophages. (A–C) THP‐1‐derived macrophages were co‐treated with AMC and/or recombinant TGF‐β1 (rTGF‐β1, 10 ng/ml). The abundance of intracellular TGF‐β1 (A), secreted TGF‐β1 (B), and M2 markers (C) was measured using qRT‐PCR and ELISA, respectively. qRT‐PCR results were normalized to GAPDH. (D) Macrophages were co‐treated with miR‐296‐5p overexpression and/or rTGF‐β1, and the expression of M2 markers was determined by qRT‐PCR. (E–G) After incubation of HCT116 and SW480 cells with CM from macrophages treated with rTGF‐β1 and AMC, EMT marker expression (E), cell motility (F), and invasiveness (G) were assessed by western blotting, transwell migration (F, scale bar 100 µm), and Matrigel invasion assays (G, scale bar 100 µm), respectively. (H‐J) In a xenograft mouse model established with parental and IR‐resistant HCT116 cells with AMC, CD206 abundance (H) in tumor tissue, the abundance of M2 markers (I), and secreted TGF‐β (J) in plasma were confirmed by IHC, qRT‐PCR, and ELISA, respectively. (K) TGF‐β mRNA levels in the plasma of CRC patients with or without lymph node metastasis were determined by qRT‐PCR. (no metastasis, n = 24; with metastasis, n = 24) The data are presented as the mean ± SD for triplicate experiments. ^*^ *p* < 0.05, ^**^ *p* < 0.01, and ^***^ *p* < 0.001 were based on Student's t‐Test for K or One‐Way ANOVA for A, B, C, D, F, G, I, and J, respectively. (L) A schematic cartoon illustrates that AMC, which regulates the circRNF10/miR‐296‐5p/Snail axis in radioresistant CRC cells, suppresses tumorigenicity by inhibiting M2 polarization and TGF‐β1 secretion in macrophages (http://biorender.io).

For in vivo validation, we examined the abundance of M2 polarization markers and TGF‐β1 in tumor tissues and plasma from mice injected with parental cells and radioresistant CRC cells, respectively. First, using immunohistochemistry (IHC) staining, we confirmed a significant increase in CD206‐positive macrophages in tumors derived from radioresistant CRC cells (Figure 6H). In addition, in tumor tissues from mice injected with radioresistant HCT116 cells, levels of M2 polarization markers (CD206, CD163, and CD11b) and TGF‐β1 were increased compared to the parental cell group, but were significantly reduced by AMC treatment (Figure 6H,I). Consistent results were also observed in plasma samples (Figure 6J). Additionally, we found elevated TGF‐β1 levels in the plasma of patients with lymph node metastasis compared to those without metastasis (Figure 6K). These results suggest that AMC upregulates miR‐296‐5p, thereby reducing macrophage M2 polarization and TGF‐β1 secretion, ultimately reducing tumor malignancy.

In summary, radiation exposure increases circRNF10 expression in CRC, leading to miR‐296‐5p suppression and upregulation of Snail, ultimately promoting malignant transformation. In contrast, AMC suppresses tumor malignancy by upregulating miR‐296‐5p and inhibiting its target, Snail. Furthermore, miR‐296‐5p translocated to macrophages within the tumor microenvironment suppresses tumor malignancy by inhibiting M2 polarization and TGF‐β1 secretion (Figure 6L). Therefore, this study suggests the potential of AMC as a therapeutic agent for radioresistant CRC and identifies the circRNF10/miR‐296‐5p/Snail axis, an AMC‐induced mechanism, as a potential diagnostic marker for radioresistant CRC.

## 3 Discussion

Although radiotherapy plays a crucial role in the treatment of locally advanced CRC, its clinical effectiveness is often limited due to intrinsic and acquired radioresistance, which impairs local tumor control and leads to recurrence and metastasis [25]. In this context, radioresistant tumor cells often acquire aggressive phenotypes such as EMT, increased migratory ability, invasiveness, and stemness, which worsen treatment outcomes and can promote disease progression after irradiation [3]. In addition, the tumor microenvironment, including cancer‐associated fibroblasts, immune cells, and hypoxic regions, creates a supportive niche that promotes tumor adaptation and resistance, further reducing the effectiveness of radiotherapy [25]. Therefore, overcoming radioresistance is a critical unmet clinical need in the treatment of CRC, and the development of effective radiosensitizing strategies and predictive biomarkers is essential to improve therapeutic responses and reduce the risk of recurrence.

In the context of oncology drug development, drugs that overcome resistance are defined not by their ability to act only on resistant cells without damaging sensitized cells, but by their ability to restore or enhance therapeutic responsiveness in tumors that do not respond to standard treatment. Most existing anticancer therapies are difficult to treat because they maintain activity in treatment‐naive or responsive tumors but lose their anticancer efficacy when the environment shifts to resistance [26]. Therefore, drugs that maintain or enhance anticancer efficacy in resistance models can be considered a significant advance in anticancer therapy. In this study, AMC significantly inhibited tumor growth in both parental (HCT116) and radioresistant CRC cell (HCT116R) xenograft models, and notably maintained antitumor activity even in radioresistant models where the response to radiation is reduced (Figure 1E,F). These results suggest that AMC is not inhibited by radioresistance‐associated pathways and can address the underlying mechanisms of radioresistance.

Drug repurposing reduces development time and cost compared with traditional drug discovery pipelines, leading to clinical application [27]. As demonstrated by the successful repurposing of thalidomide from a sedative to a key therapeutic agent for multiple myeloma [28], and the investigation of metformin for its anticancer effects beyond diabetes management [29], drug repurposing represents a promising strategy for overcoming treatment resistance in oncology. Therefore, in this study, we utilized FDA‐approved compounds to identify potential repurposed anticancer agents capable of overcoming radioresistance in CRC.

Amcinonide (AMC), among FDA‐approved compounds, was selected as an agent that effectively suppressed EMT‐related factor expression and cell viability in radioresistant CRC cells (Figure S1). This drug is a topical corticosteroid traditionally used to treat skin diseases [12]. We demonstrated that AMC suppresses tumorigenicity in radioresistant CRC cells by modulating the circRNF10/miR‐296‐5p/Snail signaling pathway. These findings were validated in various experimental models, including CRC cell lines, mouse xenograft models, and PDOs, and were further supported by clinical correlation analysis using CRC patient plasma samples.

Recent reports have shown that ncRNAs, particularly circRNAs and miRNAs, play a crucial role in modulating radiotherapy response [30]. These molecules often function within a competitive endogenous RNA (ceRNA) network, where circRNAs act as sponges to sequester miRNAs, thereby regulating the expression of downstream oncogenes involved in DNA damage repair, apoptosis, and EMT [31, 32]. CircRNAs such as circ‐MFN2 [9] and circCCDC66 [33] are known to be associated with radioresistance in CRC; however, the specific regulatory network that drives radiation‐induced EMT remains poorly understood. This study addresses this gap by elucidating the circRNF10/miR‐296‐5p axis, which mediates radioresistance in CRC cells. Specifically, we demonstrate that circRNF10, acting as a ceRNA, sponges miR‐296‐5p, thereby preventing its inhibitory targeting of Snail. These results not only broaden the current understanding of ncRNA‐mediated radioresistance mechanisms, but also suggest that the circRNF10/miR‐296‐5p axis is regulated by AMC and is a potential therapeutic target for overcoming radioresistance.

Meanwhile, the precise mechanism by which AMC regulates the expression of miR‐296‐5p has not yet been fully elucidated. Given that AMC functions as an agonist for the human GR, a ligand‐activated transcription factor [16], it is possible that GR regulates these target genes through direct promoter binding. Bioinformatics analysis using the AliBaba 2.1 tool supports this hypothesis by confirming the presence of multiple NR3C1 (gene coding for GR) binding sites within the promoter regions of miR‐296‐5p and RNF10. In particular, this study successfully verified, through luciferase reporter analysis, that GR binds to sites 5 and 6 within the RNF10 promoter region to directly regulate RNF10 transcription (Figure S8). Meanwhile, ChIP‐seq analysis suggested the possibility of potential NR3C1 binding sites (−40 ∼ −49, −306 ∼ −317) within the miR‐296‐5p promoter; however, further experimental verification is required to definitively confirm the direct transcriptional regulation of miR‐296‐5p by GR activated by AMC.

Data from CRC‐PDOs, which closely replicate individual patient tumor responses, further reinforce the clinical significance of the findings of this study. The observation that AMC suppresses Snail levels and restores radiosensitivity in RR‐PDOs (Figure 1M) suggests its potential application in personalized CRC therapy.

Furthermore, comparisons of circRNF10, miR‐296‐5p, and Snail expression patterns in the plasma of CRC patients demonstrate their potential as non‐invasive liquid biopsy biomarkers. The stability of these circulating non‐coding RNAs may be attributed to their encapsulation within tumor‐derived exosomes [34], which protects the RNA from RNase‐mediated degradation and facilitates its transport through blood circulation [35]. Consistent with this model, the in vitro analysis results of this study show that circRNF10 and miR‐296‐5p are abundant in CRC cell‐derived exosomes, supporting their existence in a protected and functionally important state. This exosome‐mediated packaging provides a valid mechanistic basis for the stable detection of these molecules in plasma samples obtained from both clinical cohorts and animal models. Long‐term monitoring of these ncRNA features can help predict response to radiotherapy and identify patients at high risk of relapse who may benefit from adjuvant therapies such as AMC.

While the results of this study highlight the therapeutic potential of AMC, it is necessary to discuss several important considerations regarding the clinical use of corticosteroids. Corticosteroids, including AMC, are potent GR agonists with well‐known immunomodulatory and metabolic effects. Chronic or systemic administration is associated with a wide range of side effects, including immunosuppression, hyperglycemia, osteoporosis, adrenal suppression, and increased susceptibility to infection [36]. These systemic effects raise potential concerns regarding the long‐term use of AMC, particularly in cancer patients undergoing concomitant radiotherapy. This is because immune function and tissue repair mechanisms are critical for therapeutic efficacy and recovery in radiotherapy [37]. Considering these points, future research should focus on optimizing dosage, duration of treatment, and route of administration to maximize the radiosensitization effect while minimizing systemic toxicity. To address these limitations in clinical application, further research appears necessary to validate potential formulation optimization strategies for AMC encapsulation utilizing advanced targeted drug delivery systems, such as recently reported tumor‐specific nanoparticles, liposomes, or oral formulations targeting CRC [38]. It is hypothesized that such advanced pharmacoengineering approaches could maximize the local bioavailability of AMC only within the microenvironment of CRC while minimizing systemic exposure. If possible, this could significantly improve the clinical safety profile while effectively maintaining potent efficacy in overcoming radioresistance.

In conclusion, this study reveals that the circRNF10/miR‐296‐5p/Snail axis is a critical driver of radioresistance in CRC and identifies AMC as a promising therapeutic modulator of this pathway. These findings, integrating ncRNA biology with drug repurposing strategies, provide strong preclinical evidence supporting the potential of AMC to enhance radiotherapy efficacy, and suggest that components of this axis could serve as predictive biomarkers for radiotherapy response in CRC patients.

## 4 Materials and Methods

### 4.1 Cell Culture

Human CRC cell lines HCT116 and SW480, and human monocyte cell line THP‐1 cells were obtained from the Korea Cell Line Bank. Cells were cultured in RPMI‐1640 medium supplemented with 10% fetal bovine serum (FBS) and 1% penicillin‐streptomycin at 37°C in a humidified incubator with 5% CO_2_. Radioresistant cells, HCT116R and SW480R, were established by irradiating 15 doses of 5 Gy (total 75 Gy), and their radioresistance characteristics have been described in a previous study [11]. HIEC‐6 cells were incubated in OptiMEM I Reduced Serum Medium supplemented with 20 mm HEPES, 10 mm GlutaMAX, 10 ng/mL epidermal growth factor (EGF), and 4% fetal bovine serum (FBS). Human umbilical vein endothelial cells (HUVECs) were cultured in CEFOgro Human Umbilical Vein Endothelial Growth Medium. All cell lines were tested for mycoplasma infection using the e‐Myco Mycoplasma PCR Detection Kit before use in the experiment.

Conditioned medium (CM) was obtained after overexpressing or treating CRC cells inoculated in 60 mm culture dishes with miR‐296‐5p, anti‐296‐5p, circRNF10, or AMC. The next day, residual serum was removed by washing three times with phosphate buffer (PBS), and the medium was replaced with fresh RPMI. After 48 h of incubation, the culture supernatant (CM) was harvested. To completely remove cell debris, the collected CM was centrifuged at 1200 rpm for 5 min at 4°C. THP‐1 cells treated with 100 ng/mL PMA were cultured for 48 h in a medium mixed with CM and fresh conditioned medium in a 1:1 ratio, after which subsequent functional analysis was performed.

### 4.2 Human Samples

Patient samples used in this study were plasma from colorectal cancer patients (KRB‐2023‐I001) provided by the Radiation Biobank (KRB) of the Korea Cancer Center Hospital, Korea Institute of Radiological and Medical Sciences (KIRAMS). Patient samples used in this study were approved by the Institutional Review Board (IRB) of KIRAMS (KIRAMS 2021‐04‐002). Patient plasma samples were obtained with prior consent from all participants, in accordance with institutional ethical guidelines. Samples provided to the research team were thoroughly anonymized and de‐identified to ensure that researchers could not access personally identifiable information.

### 4.3 Organoid Culture

Patients had locally advanced rectal cancer at clinical stage T3N+ before treatment. They received neoadjuvant chemoradiation (NCRT) and were evaluated by magnetic resonance imaging (MRI) and positron emission tomography (PET) before and after treatment. Based on tumor regression grade (TRG) after NCRT, patients were classified as radiosensitive (RS) or radioresistant (RR) [39]. The isolation and culture methods and characterization of CRC patient‐derived organoids have been well described in a previous paper [39]. CRC patient‐derived organoids were cultured in DMEM/F12 medium supplemented with B27, N‐acetyl cysteine, human epidermal growth factor, human Noggin, gastrin, and A83‐01.

### 4.4 Drug Library Screening

Radioresistant CRC cells, HCT116R and SW480R, were seeded into 96‐well plates at a density of 2 × 10^4^ cells per well. Cells were treated with either 20 µm of compounds from the SCREEN‐WELL FDA‐approved drug library V2 (Enzo, #BML‐2843‐0100) or DMSO as a control and incubated for 48 h, and then harvested. mRNA expression levels of EMT markers were measured using qRT‐PCR. Amcinonide was purchased from TCI, dissolved in DMSO, and then diluted to a concentration of 80 µm in the culture medium for use.

### 4.5 Irradiation

Cells were irradiated using a 137Cs gamma‐ray source (Biobeam 8000) at a dose rate of 3.81 Gy/min. For in vitro experiments, cells received a single radiation dose of 5 Gy as indicated. For in vivo experiments, tumor‐bearing mice were anesthetized and subjected to localized irradiation with a single dose of 10 Gy.

### 4.6 Cell Viability Analysis

Cell viability was assessed using the WST‐8 cell viability assay kit according to the manufacturer's instructions. Briefly, cells were seeded in 96‐well plates at a density of 4 × 10^3^ cells per well and treatment with 20, 40, 60, 80, 100, 150, 200, 300, 350, and 400 µm or 5, 10, 20, 30, 40, 50, 60, 80, 100, 150, 200, 250, 300, and 350 µm with or without 5 Gy irradiation. After 48 h of incubation, absorbance was measured at 450 nm using a microplate reader.

### 4.7 Clonogenic Assay

Colony formation assay was performed by seeding HCT116 or HCT116 5FUR cells at densities 1 × 10^3^, 2.5 × 10^4^, 3.5 × 10^4^, 5 × 10^4^, and 7.5 × 10^4^ cells/dish and SW480 or SW480 5FUR cells at densities 5 × 10^3^, 3.0 × 10^4^, 4.5 × 10^4^, 6 × 10^4^, and 9 × 10^4^ cells/dish into 60 mm culture dishes. The following day, cells were treated with various concentrations of 5‐FU (0, 10, 20, 30, and 40 µm). After 2 weeks of incubation, colonies were fixed with methanol, stained with crystal violet, and counted.

### 4.8 Western Blotting

Cells or tumor tissues were lysed in RIPA buffer containing protease and phosphatase inhibitors. Protein concentrations were measured using a Bradford assay. Proteins were separated by SDS‐PAGE and transferred to PVDF membranes. The membranes were blocked with 5% BSA to TBS‐T and incubated with primary antibodies overnight at 4°C. Antibodies against Vimentin, Snail, and Zeb1 were purchased from Cell Signaling Technology. Antibodies against Slug and β‐actin were obtained from Santa Cruz Biotechnology. Antibody against Twist was purchased from Abcam. After incubation with HRP‐conjugated secondary antibodies, protein signals were detected using an enhanced chemiluminescence (ECL) system and visualized.

### 4.9 Quantitative Realtime‐PCR

Total RNA was extracted from cells or tissues using TRIzol. cDNA was synthesized according to the manufacturer's instructions using the cDNA Synthesis Kit and the Mir X miRNA First cDNA Synthesis Kit. qRT‐PCR was performed on a LightCycler 96 using Power SYBR Green PCR Master Mix. Relative gene expression levels were calculated using the 2^−△△Ct^ method, with GAPDH and U6 as internal controls. Primer sequences are listed in Supplementary Table S1.

### 4.10 Subcutaneous Xenograft Animal Model

All animal experimental procedures were approved by the Institutional Animal Care and Use Committee (IACUC) of the Korea Institute of Radiological Sciences (kirams2022‐0131) and were conducted in accordance with institutional and national guidelines. A suspension of 1 × 10^7^ HCT116 or HCT116R cells in Matrigel was injected subcutaneously into the right thigh of 6‐week‐old female Balb/c nude mice, respectively. One week after implantation, AMC was administered intraperitoneally at a dose of 7 mg/kg, 14 times over 3 weeks. To enhance the solubility of hydrophobic compounds and minimize solvent‐induced toxicity, AMC was dissolved in a co‐solvent vehicle consisting of 5% DMSO, 40% PEG300, 5% Tween‐80, and 50% sterile saline. A vehicle control group, administered the identical solvent mixture without AMC, was maintained under the same conditions to control for any baseline vehicle effects. 28 days after implantation, the tumors were excised, weighed, and processed for histological and molecular analyses. Tumor volume was calculated as (width × width × length)/2.

### 4.11 Xenograft Animal Model With Combined IR and AMC

Six‐week‐old female Balb/c nude mice were subcutaneously injected in the right thigh with 1 × 10^7^ HCT116 or HCT116R cells suspended in Matrigel. One week later, the tumors were partially irradiated with 10 Gy, followed by intraperitoneal administration of AMC at 7 mg/kg seven times over 2 weeks. Fifteen days after treatment, the tumors were excised, weighed, and processed for histological and molecular analysis. Tumor volume was calculated as (width × width × length)/2.

### 4.12 Transwell Migration and Invasion Assays

For the migration and invasion assays, the bottom surface of the Transwell inserts with 8 µm pores was coated with 0.2% gelatin. For invasion analysis, the upper surface of the Transwell membrane was coated with Matrigel. HCT116 cells (migration, 5 × 10^5^ cells/well; invasiveness, 1 × 10^6^ cells/well) or SW480 cells (migration, 3 × 10^5^ cells/well; invasiveness, 3 × 10^5^ cells/well) (including parental or radioresistant cells) were seeded into the upper chamber of a Transwell insert. After cells were incubated 16 h, the transwell was fixed with methanol and stained with crystal violet. The graph was generated based on the quantified number of cells. Cell counting was performed using ImageJ software, and the graph was created based on the results.

### 4.13 Luciferase Reporter Assay

The predicted binding sites between miR‐296‐5p and target‐related candidate proteins (Snail, circRNF10, and TGF‐β1) were predicted using an online bioinformatics database. To confirm direct binding, wild‐type (WT) or mutant (MUT) sequences containing the predicted binding sites were synthesized and cloned into the pmirGLO luciferase reporter vector. For reporter analysis, HCT116 cells were seeded into 24‐well plates at a cell density of 1 × 10^5^ per well and cultured overnight. Subsequently, the cells were co‐transfected with the WT or MUT reporter and the miR‐296‐5p mimic or negative control (NC) using Lipofectamine 2000 according to the manufacturer's instructions.

After 48 h post‐transfection, cells were washed with PBS and harvested using passive lysis buffer. Luciferase activity was measured sequentially using a Dual‐Luciferase Reporter Assay System on a luciferase assay microplate reader. Firefly luciferase activity was normalized to Renilla luciferase activity to correct for transformation efficiency. All experiments were performed in triplicate.

### 4.14 Treatment With RNase R

Total RNA was isolated from cells using the indicated RNA extraction method. For RNase R digestion, 1 µg of total RNA was incubated with RNase R in an appropriate reaction buffer at 37°C for 30 min according to the manufacturer's instructions. After digestion and RNA purification, the RNA was reverse transcribed, and RNF10 mRNA and circRNF10 were analyzed by qRT‐PCR using primers.

### 4.15 Treatment With Actinomycin D

To evaluate RNA stability, cells were seeded into 24‐well plates and treated with the indicated concentration of Actinomycin D to inhibit de novo transcription. Cells were harvested at 4, 8, and 16 h after treatment, and RNA was extracted. The isolated RNA samples were treated with DNase I and reverse transcribed to generate cDNA. Transcript levels were subsequently measured by qRT‐PCR. RNA abundance at each time point was normalized to an internal control, and RNA half‐life was estimated based on the remaining RNA relative to the 0‐h time point.

### 4.16 ELISA of TGF‐β1

TGF‐β1 levels were measured using a commercially available ELISA kit according to the manufacturer's instructions. Briefly, standards and samples were added to pre‐coated 96‐well plates, followed by incubation with the detection antibody and substrate development. Absorbance was measured with a microplate reader, and TGF‐β1 was calculated from the standard curve and expressed as pg/mL.

### 4.17 Fluorescence Imaging Analysis

CRC cells were transformed with miR‐296‐5p tagged with cy3 using G‐fectin, and CM was obtained 48 h after the medium was changed the following day. Macrophages differentiated on THP‐1 by treatment with 100 ng/mL PMA were cultured in the added CM, and the cells were fixed with a 4% paraformaldehyde solution. Cell morphology was confirmed using a bright‐field microscope, and the fluorescence of cy3 expressed within the cells was measured using a fluorescence microscope.

### 4.18 Cloning, Transfection, and Reagents

circRNF10 was inserted into the pcDNA3.1(+) circRNA mini vector, and the Snail overexpression gene was inserted into the pcDNA3.1 vector. The primer sequences used for cloning are listed in Supplementary Table S1.

Plasmids, miRNAs, and siRNAs were transformed into cells using Lipofectamine 2000 reagent or G‐fectin according to the manufacturer's instructions.

THP‐1 cells differentiated by PMA treatment were treated with 10 ng/mL of recombinant TGF‐β1 (rTGF‐β1), and the indicated experiments were performed. CRC cells were treated with RU‐486 (10 µm), Actinomycin D (10 µg/mL), or GW4869 (10 µM) in the culture medium, respectively, and the corresponding experiments were performed.

### 4.19 Exosome Isolation and Identification

Exosomes were isolated from CRC cell line culture medium (CM) using the ExoQuick‐TC kit according to the manufacturer's protocol. Briefly, the CRC cell culture medium was centrifuged to remove cellular debris, and the ExoQuick‐TC solution was added to the supernatant. After incubating the mixture overnight at 4°C, the supernatant was removed by centrifugation, and the exosome pellet was collected. For Western blot analysis of exosome marker expression, the exosome pellet was resuspended in RIPA buffer supplemented with a protease inhibitor, and proteins were isolated. Exosome RNA was extracted using the SeraMir Exosome RNA Purification Column Kit according to the manufacturer's instructions.

### 4.20 Promoter Luciferase Assay

To construct the reporter plasmids, the WT and MUT NR3C1 binding sites of the RNF10 promoter were cloned into the pGL3‐basic vector. HCT116 cells were co‐transfected with either the WT or MUT construct along with the pRL‐CMV‐Renilla plasmid using Lipofectamine 2000, followed by treatment with or without 80 µm AMC. Luciferase activity was determined using the Dual‐Luciferase Reporter Assay System according to the manufacturer's protocol and normalized to Renilla luciferase activity.

### 4.21 Flow Cytometry Analysis of Cell Death

Cell death was evaluated using PI Staining according to the manufacturer's instructions. HCT116 and SW480 cells were seeded in 6‐well plates and treated with 80 and 200 µm of AMC for 48 h. After incubation, both cell lines were harvested, washed twice with cold PBS, and resuspended in binding buffer containing PI. Staining was performed for 15 min under room temperature and dark conditions. Finally, binding buffer was added to each sample, and cell death was immediately analyzed using a flow cytometer.

### 4.22 Statistical Analysis

All data are presented as mean ± standard deviation (SD) as indicated. Spearman correlation and statistical analyses were performed using GraphPad Prism version 8 (GraphPad Software, USA). Comparisons between two groups were made using unpaired two‐tailed Student's t‐tests, and comparisons among multiple groups were performed using one‐way ANOVA. A *p*‐value < 0.05 was considered statistically significant.

## Author Contributions

Conceptualization was carried out by I.H.B.; methodology by H.J.S., D.H.L., J.H.K. and U.S.S.; data curation by H.J.S.; investigation by H.J.S. and D.H.L.; the original draft was written by H.J.S.; review and editing were done by I.H.B.; funding was acquired by I.H.B.; project administration was handled by I.H.B.; and supervision was provided by I.H.B

## Conflicts of Interest

The authors declare no conflicts of interest.

## Supporting information




**Supporting File 1**: advs78502‐sup‐0001‐SuppMat.pdf.

**Supporting File 2**: advs78502‐sup‐0002‐SuppMat.docx.

## Data Availability

The data that support the findings of this study are available from the corresponding author upon reasonable request.

## References

[1] R. Sauer , H. Becker , W. Hohenberger , et al., “Preoperative Versus Postoperative Chemoradiotherapy for Rectal Cancer,” New England Journal of Medicine 351, no. 17 (2004): 1731–1740, 10.1056/NEJMoa040694. 15496622

[2] Y. Li , J. Wang , X. Ma , et al., “A Review of Neoadjuvant Chemoradiotherapy for Locally Advanced Rectal Cancer,” International Journal of Biological Sciences 12, no. 8 (2016): 1022–1031, 10.7150/ijbs.15438. PMC4971740 27489505

[3] L. Qiao , Y. Chen , N. Liang , et al., “Targeting Epithelial‐to‐Mesenchymal Transition in Radioresistance: Crosslinked Mechanisms and Strategies,” Frontiers in Oncology 12, no. 1 (2022): 1–14, 10.3389/fonc.2022.775238. PMC8888452 35251963

[4] Y. Xia , M. Sun , H. Huang , and W. L. Jin , “Drug Repurposing for Cancer Therapy,” Signal Transduction and Targeted Therapy 9, no. 1 (2024): 1–33, 10.1038/s41392-024-01808-1. PMC11026526 38637540

[5] A. Kirtonia , K. Gala , S. G. Fernandes , et al., “Repurposing of Drugs: An Attractive Pharmacological Strategy for Cancer Therapeutics,” Seminars in Cancer Biology 68, no. 1 (2021): 258–278, 10.1016/j.semcancer.2020.04.006. 32380233

[6] C. H. Schein , “Repurposing Approved Drugs for Cancer Therapy,” British Medical Bulletin 137, no. 1 (2021): 13–27, 10.1093/bmb/ldaa045. PMC7929227 33517358

[7] C. M. Telleria , “Drug Repurposing for Cancer Therapy,” Journal of Cancer Science & Therapy 4, no. 7 (2012): 1–5, 10.4172/1948-5956.1000e108. PMC3440183 22984635

[8] Q. Yu , W. Zhang , X. Zhou , W. Shen , C. Xing , and X. Yang , “Regulation of lnc‐TLCD2‐1 on Radiation Sensitivity of Colorectal Cancer and Comprehensive Analysis of Its Mechanism,” Frontiers in Oncology 11, no. 1 (2021): 1–14, 10.3389/fonc.2021.714159. PMC8320535 34336703

[9] D. Liu , S. Peng , Y. Li , and T. Guo , “Circ‐MFN2 Positively Regulates the Proliferation, Metastasis, and Radioresistance of Colorectal Cancer by Regulating the miR‐574‐3p/IGF1R Signaling Axis,” Frontiers in Genetics 12, no. 1 (2021): 1–11, 10.3389/fgene.2021.671337. PMC8170135 34093664

[10] L. Li , Z. Jiang , X. Zou , and T. Hao , “Exosomal circ_IFT80 Enhances Tumorigenesis and Suppresses Radiosensitivity in Colorectal Cancer by Regulating miR‐296‐5p/MSI1 Axis,” Cancer Management and Research 13, no. 1 (2021): 1929–1941, 10.2147/CMAR.S297123. PMC7917334 33658855

[11] J. Y. Choi , H. J. Seok , D. H. Lee , et al., “miR‐1226‐5p is Involved in Radioresistance of Colorectal Cancer by Activating M2 Macrophages Through Suppressing IRF1,” Journal of Translational Medicine 22, no. 1 (2024): 1–19, 10.1186/s12967-024-05797-1. PMC11523791 39472937

[12] R. Hayakawa , K. Matsunaga , M. Suzuki , and K. Hosokawa , “Contact Dermatitis From Amcinonide,” Contact Dermatitis 16, no. 1 (1987): 48–49, 10.1111/j.1600-0536.1987.tb02627.x. 3816214

[13] L. Thorel , M. Perréard , R. Florent , et al., “Patient‐Derived Tumor Organoids: A New Avenue for Preclinical Research and Precision Medicine in Oncology,” Experimental & Molecular Medicine 56, no. 7 (2024): 1531–1551, 10.1038/s12276-024-01272-5. PMC11297165 38945959

[14] J. Hayes , P. P. Peruzzi , and S. Lawler , “MicroRNAs in Cancer: Biomarkers, Functions and Therapy,” Trends in Molecular Medicine 20, no. 8 (2014): 460–469, 10.1016/j.molmed.2014.06.005. 25027972

[15] Q. Shang , Z. Yang , R. Jia , and S. Ge , “The Novel Roles of circRNAs in Human Cancer,” Molecular Cancer 18, no. 1 (2019): 1–10, 10.1186/s12943-018-0934-6. PMC6325800 30626395

[16] E. R. Weikum , M. T. Knuesel , E. A. Ortlund , and K. R. Yamamoto , “Glucocorticoid Receptor Control of Transcription: Precision and Plasticity Via Allostery,” Nature Reviews Molecular Cell Biology 18, no. 3 (2017): 159–174, 10.1038/nrm.2016.152. PMC6257982 28053348

[17] K. L. Kolb , A. L. S. Mira , E. D. Auer , et al., “Glucocorticoid Receptor Gene (NR3C1) Polymorphisms and Metabolic Syndrome: Insights from the Mennonite Population,” Genes 14, no. 9 (2023): 1–18, 10.3390/genes14091805. PMC10530687 37761945

[18] H. E. Barker , J. T. Paget , A. A. Khan , and K. J. Harrington , “The Tumour Microenvironment After Radiotherapy: Mechanisms of Resistance and Recurrence,” Nature Reviews Cancer 15, no. 7 (2015): 409–425, 10.1038/nrc3958. PMC4896389 26105538

[19] F. Zhang , H. Wang , X. Wang , et al., “TGF‐β Induces M2‐Like Macrophage Polarization via SNAIL‐Mediated Suppression of a Pro‐Inflammatory Phenotype,” Oncotarget 7, no. 32 (2016): 52294–52306, 10.18632/oncotarget.10561. PMC5239552 27418133

[20] S. Wang , J. Shu , N. Wang , and Z. He , “Exosomal Non‐Coding RNAs: Mediators of Crosstalk Between Cancer and Cancer Stem Cells,” Cell Death Discovery 11, no. 1 (2025): 1–16, 10.1038/s41420-025-02726-z. PMC12501049 41053039

[21] M. Luo , F. Zhao , H. Cheng , M. Su , and Y. Wang , “Macrophage Polarization: An Important Role in Inflammatory Diseases,” Frontiers in Immunology 15, no. 1 (2024): 1–10, 10.3389/fimmu.2024.1352946. PMC11039887 38660308

[22] D. Gong , W. Shi , S.‐J. Yi , H. Chen , J. Groffen , and N. Heisterkamp , “TGFβ Signaling Plays a Critical Role in Promoting Alternative Macrophage Activation,” BMC Immunology 13, no. 1 (2012): 1–10, 10.1186/1471-2172-13-31. PMC3406960 22703233

[23] J. Cai , L. Xia , J. Li , S. Ni , H. Song , and X. Wu , “Tumor‐Associated Macrophages Derived TGF‐β‒Induced Epithelial to Mesenchymal Transition in Colorectal Cancer Cells Through Smad2,3‐4/Snail Signaling Pathway,” Cancer Research and Treatment 51, no. 1 (2019): 252–266, 10.4143/crt.2017.613. PMC6333993 29690747

[24] Z. Liu , W. Kuang , Q. Zhou , and Y. Zhang , “TGF‐β1 Secreted by M2 Phenotype Macrophages Enhances the Stemness and Migration of Glioma Cells via the SMAD2/3 Signalling Pathway,” International Journal of Molecular Medicine 42 (2018): 3395–3403, 10.3892/ijmm.2018.3923. PMC6202079 30320350

[25] Y. Zhong , X. Chen , S. Wu , et al., “Deciphering Colorectal Cancer Radioresistance and Immune Microrenvironment: Unraveling the Role of EIF5A Through Single‐Cell RNA Sequencing and Machine Learning,” Frontiers in Immunology 15, no. 1 (2024): 1–15, 10.3389/fimmu.2024.1466226. PMC11405197 39290702

[26] N. Vasan , J. Baselga , and D. M. Hyman , “A View on Drug Resistance in Cancer,” Nature 575, no. 7782 (2019): 299–309, 10.1038/s41586-019-1730-1. PMC8008476 31723286

[27] S. Pushpakom , F. Iorio , P. A. Eyers , et al., “Drug Repurposing: Progress, Challenges and Recommendations,” Nature Reviews Drug Discovery 18, no. 1 (2019): 41–58, 10.1038/nrd.2018.168. 30310233

[28] G. G. Amare , B. G. Meharie , and Y. M. Belayneh , “A Drug Repositioning Success: The Repositioned Therapeutic Applications and Mechanisms of Action of Thalidomide,” Journal of Oncology Pharmacy Practice 27, no. 3 (2021): 673–678, 10.1177/1078155220975825. 33249990

[29] Y. K. Chae , A. Arya , M.‐K. Malecek , et al., “Repurposing Metformin for Cancer Treatment: Current Clinical Studies,” Oncotarget 7, no. 26 (2016): 40767–40780, 10.18632/oncotarget.8194. PMC5130043 27004404

[30] K. Xu , H. Guo , A. Xia , Z. Wang , S. Wang , and Q. Wang , “Non‐Coding RNAs in Radiotherapy Resistance: Roles and Therapeutic Implications in Gastrointestinal Cancer,” Biomedicine & Pharmacotherapy 161, no. 1 (2023): 1–16, 10.1016/j.biopha.2023.114485. 36917887

[31] X. Wang , H. Li , Y. Lu , and L. Cheng , “Circular RNAs in Human Cancer,” Frontiers in Oncology 10, no. 1 (2020): 1–14, 10.3389/fonc.2020.577118. PMC7848167 33537235

[32] M. Shahpari , M. Hashemi , T. Younesirad , A. Hasanzadeh , M. M. Mosanne , and M. Ahmadifard , “The Functional Roles of Competitive Endogenous RNA (ceRNA) Networks in Apoptosis in Human Cancers: The circRNA/miRNA/mRNA Regulatory Axis and Cell Signaling Pathways,” Heliyon 10, no. 21 (2024): 1–22, 10.1016/j.heliyon.2024.e37089. PMC11546195 39524849

[33] L. Wang , X. Peng , X. Lu , Q. Wei , M. Chen , and L. Liu , “Inhibition of hsa_circ_0001313 (circCCDC66) Induction Enhances the Radio‐Sensitivity of Colon Cancer Cells Via Tumor Suppressor miR‐338‐3p,” Pathology ‐ Research and Practice 215, no. 4 (2019): 689–696, 10.1016/j.prp.2018.12.032. 30630646

[34] J. Li , W. Zhou , H. Wang , M. Huang , and H. Deng , “Exosomal Circular RNAs in Tumor Microenvironment: An Emphasis on Signaling Pathways and Clinical Opportunities,” MedComm 5, no. 12 (2024): 1–17, 10.1002/mco2.70019. PMC11586091 39584047

[35] L. Cheng , R. A. Sharples , B. J. Scicluna , and A. F. Hill , “Exosomes Provide a Protective and Enriched Source of miRNA for Biomarker Profiling Compared to Intracellular and Cell‐Free Blood,” Journal of Extracellular Vesicles 3, no. 1 (2014): 1–14, 10.3402/jev.v3.23743. PMC3968297 24683445

[36] R. Pofi , G. Caratti , D. W. Ray , and J. W. Tomlinson , “Treating the Side Effects of Exogenous Glucocorticoids; Can We Separate the Good From the Bad?,” Endocrine Reviews 44, no. 6 (2023): 975–1011, 10.1210/endrev/bnad016. PMC10638606 37253115

[37] S. C. Formenti and S. Demaria , “Systemic Effects of Local Radiotherapy,” The Lancet Oncology 10, no. 7 (2009): 718–726, 10.1016/S1470-2045(09)70082-8. PMC2782943 19573801

[38] L. Sun , H. Liu , Y. Ye , et al., “Smart Nanoparticles for Cancer Therapy,” Signal Transduction and Targeted Therapy 8, no. 1 (2023): 1–28, 10.1038/s41392-023-01642-x. PMC10622502 37919282

[39] J. Lee , J. Kwon , D. Kim , et al., “Gene Expression Profiles Associated with Radio‐Responsiveness in Locally Advanced Rectal Cancer,” Biology 10, no. 6 (2021): 1–17, 10.3390/biology10060500. PMC8226560 34205090

