# Supplementary material for: Amcinonide Exhibits Antitumor Effects in Radioresistant Colorectal Cancer Through the circRNF10/miR‐296‐5p/Snail Axis
Source: Adv Sci (Weinh). 2026 Oct 26:e78502. Online ahead of print. doi: 10.1002/advs.78502 (PMC13710703; doi:10.1002/advs.78502)
Supplement: Supplementary file 1 [file ADVS-9999-e78502-s001.pdf]

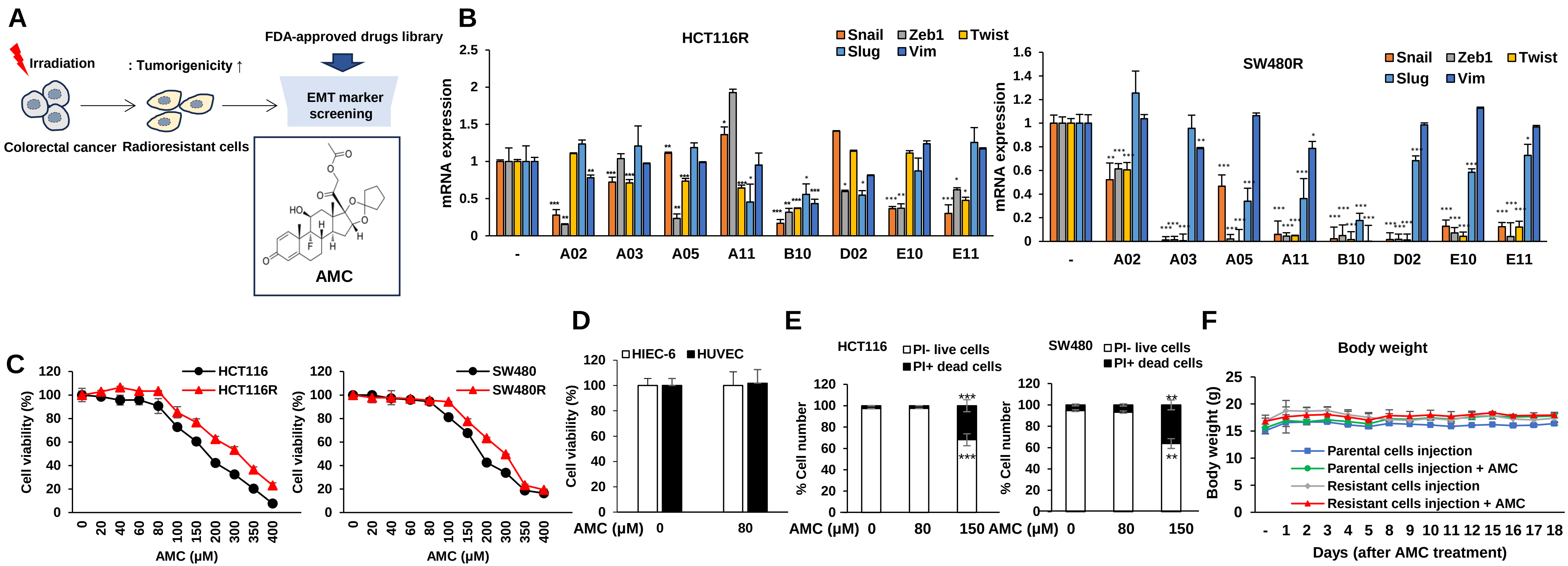

Supplementary Figure 1 | AMC, selected as a drug that significantly reduces the levels of EMT markers in radioresistant CRC cells, was used at a concentration that does not induce toxicity in normal cells and mouse experiments. (A) To screen for drugs that effectively inhibit the expression of EMT markers, FDA-approved library drugs were treated individually in established radioresistant colorectal cancer (CRC) cells. (B) After treating radioresistant HCT116R and SW480R cells with various drugs (20 μM) for 48 hours, mRNA expression of EMT markers Snail, Zeb1, Twist, Slug, and Vimentin was measured by qRT-PCR. qRT-PCR results were normalized to GAPDH. (C) Parental (HCT116 and SW480) and radioresistant cells (HCT116R and SW480R) were treated with 20, 40, 60, 80, 100, 150, 200, 300, 350, and 400 μM AMC, followed by a WST-8 viability assay. (D) After treating normal intestinal cells, HIEC-6 and HUVEC cells, with 80 μM AMC, the cell proliferation rate was evaluated using the WST-8 assay for 48 hours. (E) Flow cytometry results using PI staining were showed to confirm the cytotoxicity of HCT116 and SW480 cells treated with AMC (80 and 150 μM). (F) Changes in mouse body weight over time were measured in experiments to verify the in vivo efficacy of AMC. Data are presented as the mean ± standard deviation (SD) of triplicate experiments. *P < 0.05, **P < 0.01, ***P < 0.001 were based on Student's t-Test (B and C).

Supplementary Figure S2

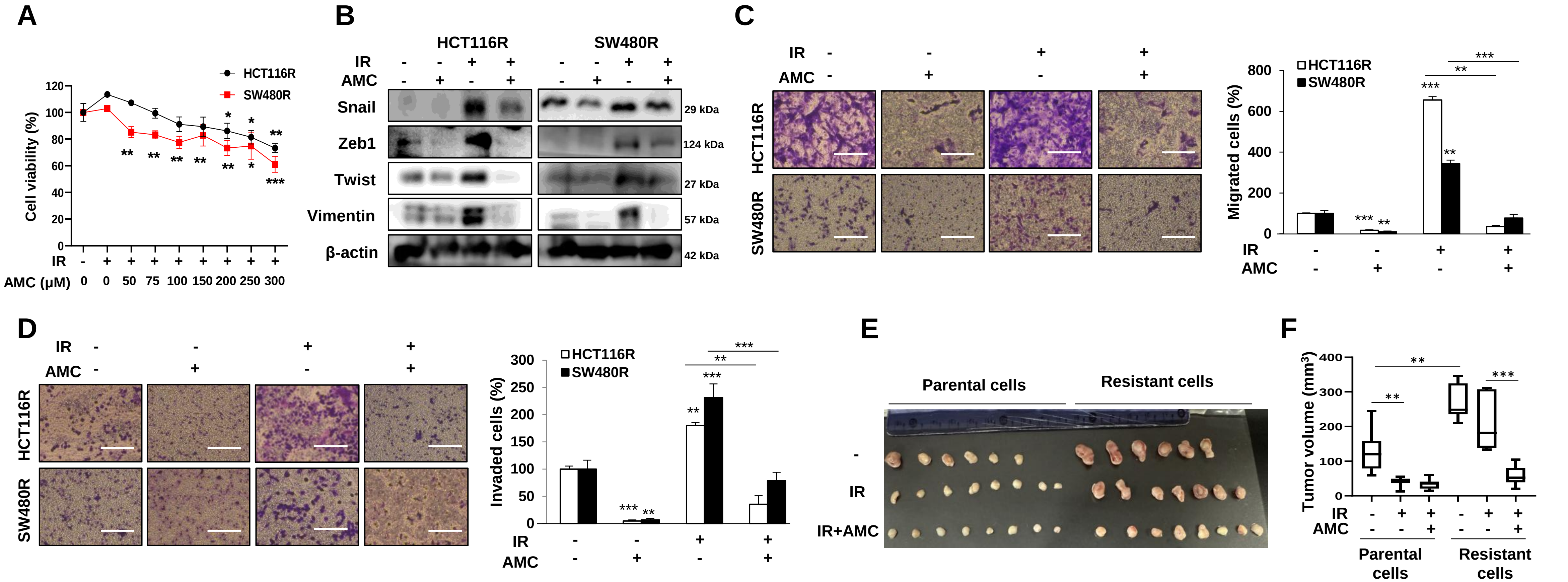

Supplementary Figure 2 | AMC enhances the radiosensitivity of radioresistant CRC cells. (A) Two radioresistant CRC cell lines were treated with 5 Gy radiation in combination with 50, 75, 100, 150, 200, 250, and 300 uM of AMC, and cell viability was assessed after 48 h using the WST-8 assay. (B-D) After co-treatment with 5 Gy radiation and 80 μM AMC, EMT marker expression (B), migratory capacity (C), and invasive ability (D) were determined by western blotting, transwell migration (C, scale bar 100 μM), and matrigel invasion assays (D, scale bar 100 μM), respectively. (E-F) Parental HCT116 and radioresistant HCT116R cells were subcutaneously injected into the right thigh of Balb/c nude mice. One week after injection, mice were exposed to 10 Gy irradiation and treated with AMC (7 mg/kg) via intraperitoneal injection seven times over 2 weeks. After 15 days, the mice were sacrificed, and the tumors were excised for analysis of tumor size (E) and volume (F) (parental cell lines: NC, n = 6; IR, n = 8; IR+AMC, n = 8; radioresistant cell lines: NC, n = 6; IR, n = 7; IR+AMC, n = 8). The data are presented as the mean ± SD for triplicate experiments. *P < 0.05, **P < 0.01, and ***P < 0.001 were analyzed using Student's t-test (A) or one-way ANOVA (C, D, and F), respectively.

Supplementary Figure S3

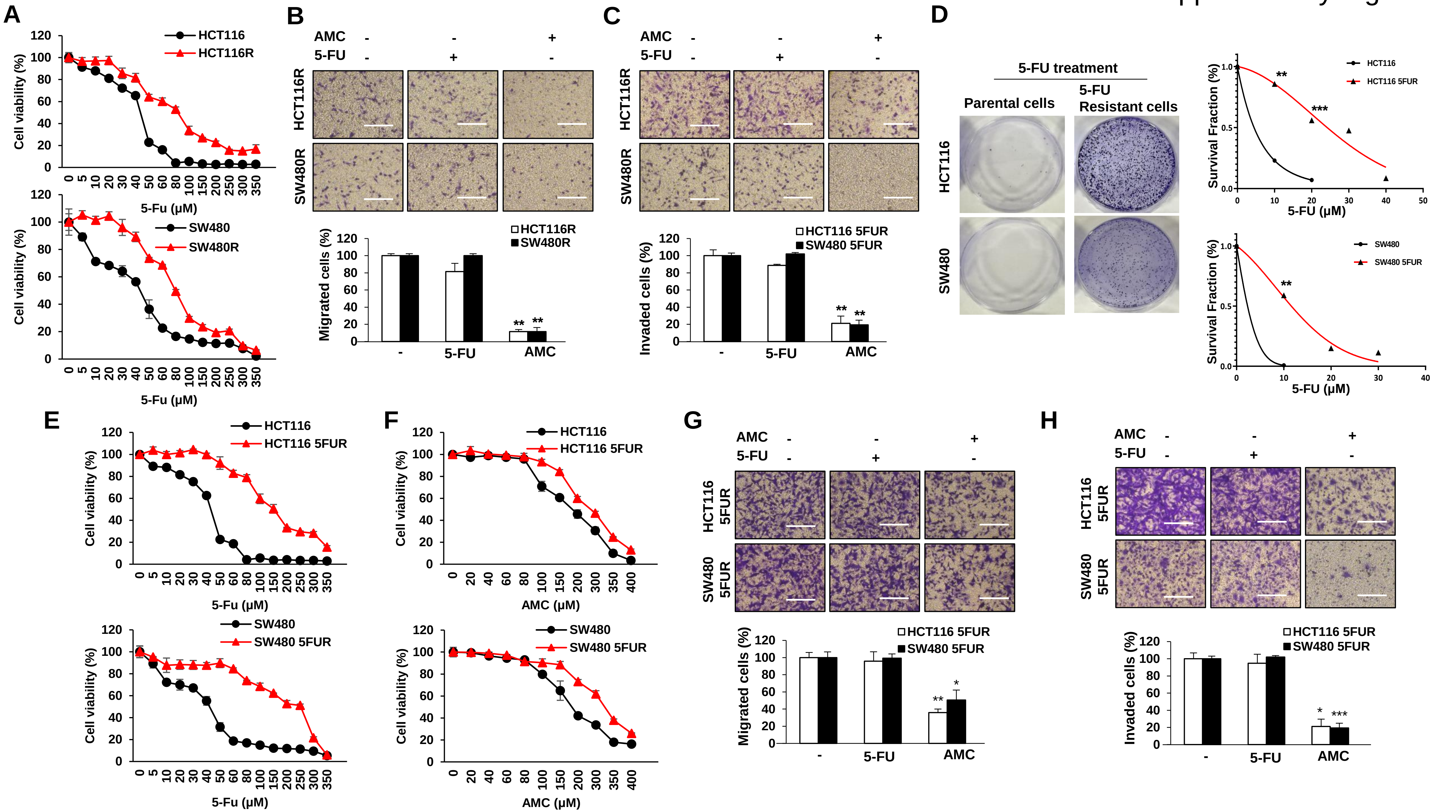

Supplementary Figure 3 | AMC exhibits greater inhibitory effects on tumorigenicity than 5-FU in radioresistant CRC cells. (A) Parental (HCT116 and SW480) and radioresistant CRC cells (HCT116R and SW480R) were treated with various concentrations (5, 10, 20, 30, 40, 50, 60, 80, 100, 150, 200, 250, 300, and 350 μM) of 5-FU, and cell viability was measured after 48 h using the WST-8 assay. (B-C) HCT116R and SW480R cells were treated with 5-FU (5 μM) and AMC (80 μM), and migratory ability (B) and invasiveness (C) were measured using transwell migration (B, scale bar 100 μM) and matrigel invasion (C, scale bar 100 μM) assays, respectively. (D) Representative images of colony formation following treatment with 5-FU (10 μM) are shown. The graph presents the results of a colony formation assay performed in parental (HCT116 and SW480) and the 5-FU-resistant CRC cells (HCT116 5FUR and SW480 5FUR) treated with various concentrations of 5-FU (0, 10, 20, 30, and 40 μM). (E) Parental and 5-FU-resistant CRC cells were treated with various concentrations of 5-FU (5, 10, 20, 30, 40, 50, 60, 80, 100, 150, 200, 250, 300, and 350 μM), and cell viability was measured after 48 h using the WST-8 assay. (F) Parental (HCT116 and SW480) and 5-FU-resistant CRC cells (HCT116 5FUR and SW480 5FUR) were treated with various concentrations (20, 40, 60, 80, 100, 150, 200, 300, 350, and 400 μM) of AMC, and cell viability was assessed 48 h using the WST-8 assay. (G-H) HCT116 5FUR and SW480 5FUR CRC cells were treated with 5-FU (60 μM) or AMC (80 μM), and migration (G) and invasion (H) were measured using transwell migration (G, scale bar 100 μM) and matrigel invasion (H, scale bar 100 μM) assays, respectively. The data are presented as the mean ± SD for triplicate experiments. $^{*}P < 0.05$, $^{**}P < 0.01$, and $^{***}P < 0.001$ were based on Student's t-Test (A, D, E, and F) or One-Way ANOVA (B, C, G, and H), respectively.

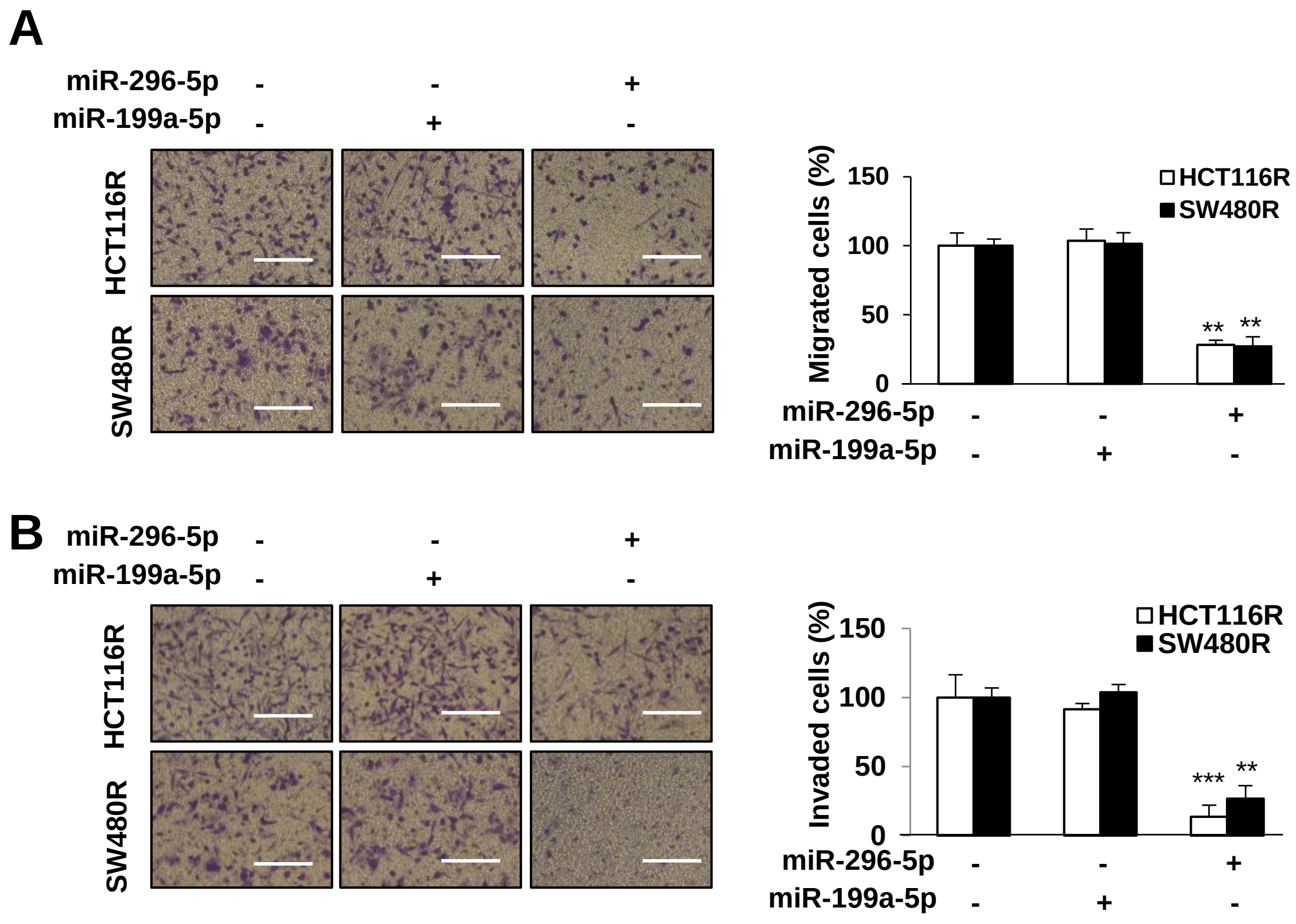

Supplementary Figure 4 | miR-296-5p inhibits the tumorigenicity of radioresistant CRC cells. (A-B) After transfecting HCT116R and SW480R cells with miR-296-5p and miR-199a-5p mimics, migration ability (A) and invasiveness (B) were measured using transwell migration (A, scale bar 100 μm) and matrigel invasion (B, scale bar 100 μm) assays, respectively. The graph was constructed by counting the number of cells in the migration and invasion images using Image J. The data are presented as the mean ± SD for triplicate experiments. *P < 0.05, **P < 0.01, and ***P < 0.001 were analyzed using One-Way ANOVA for B and C.

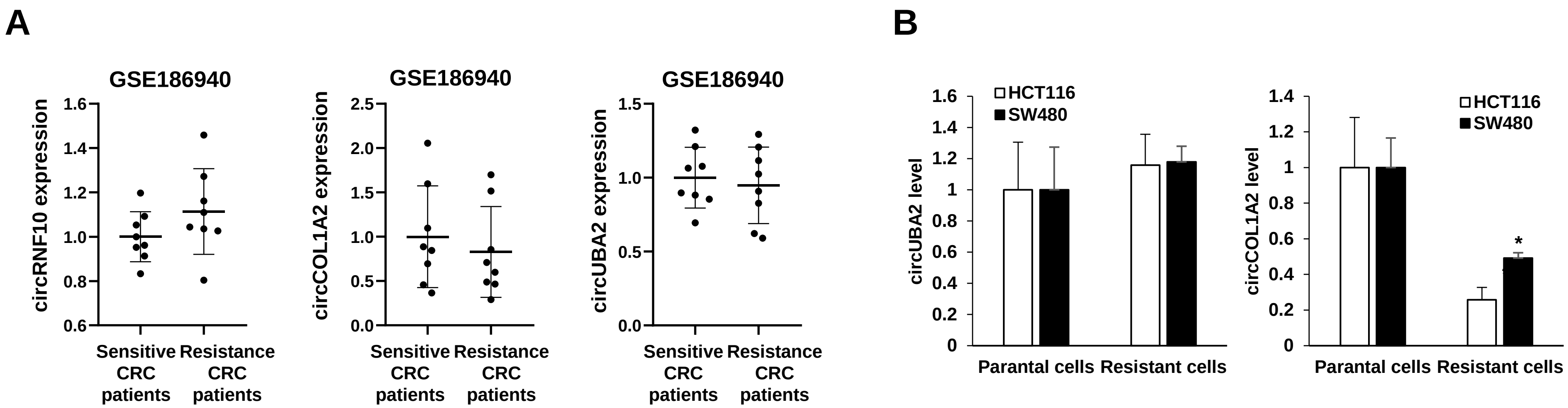

Supplementary Figure 5 | circRNF10 is upregulated in radioresistant CRC patients and cell lines compared to radiosensitive CRC patients and cell lines. (A) Using the GEO database (GSE186940) for radiosensitive and radioresistant CRC tissues, the levels of miR-296-5p-targeting circRNAs, circRNF10, circCOL1A2, and circUBA2. (B) The levels of circCOL1A2 and circUBA2 in parental and radioresistant HCT116 and SW480 cells using qRT-PCR. qRT-PCR results were normalized to GAPDH. Data are presented as the mean ± standard deviation (SD) of triplicate experiments. *P < 0.05, **P < 0.01, ***P < 0.001 were based on Student’s t-Test for B.

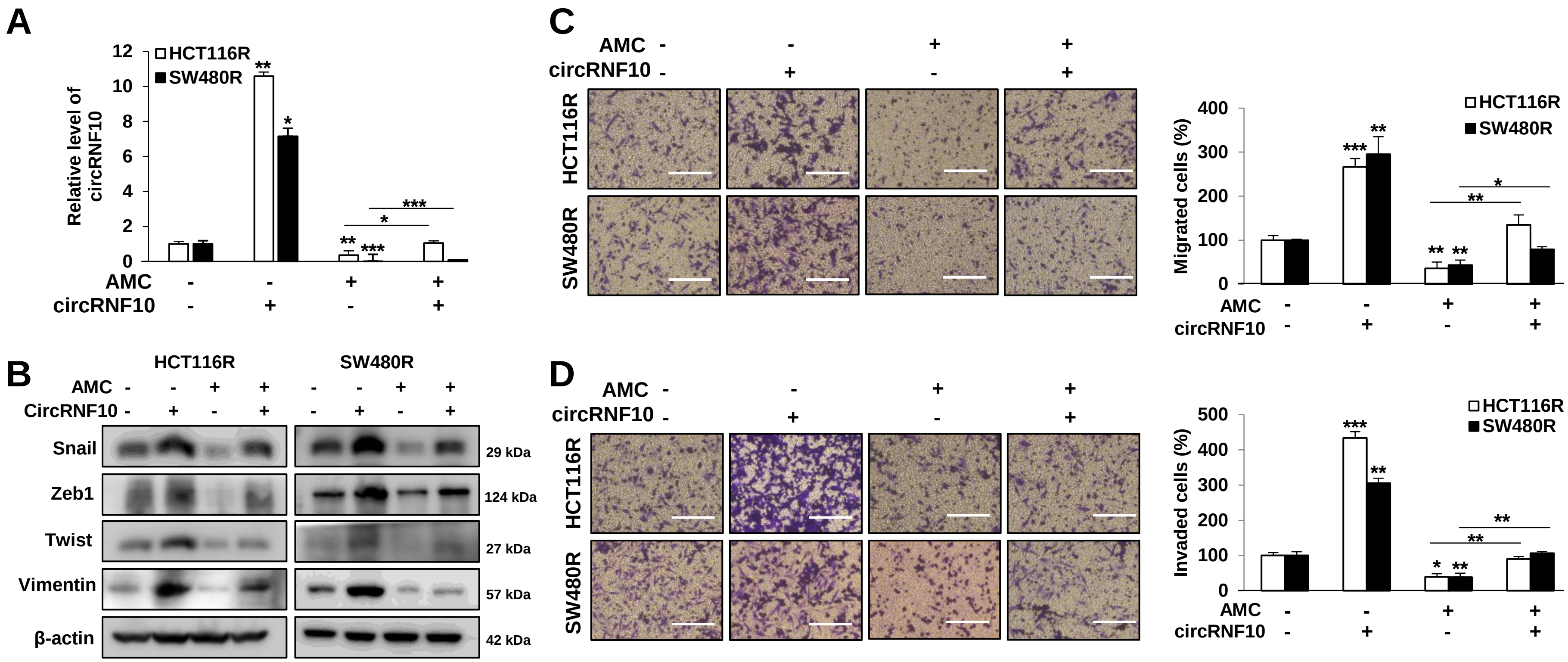

Supplementary Figure 6 | AMC inhibits the mechanism of tumor malignancy by inhibiting circRNF10. (A-D) After treating circRNF10-overexpressing HCT116R and SW480R cells with AMC, circRNF10 mRNA levels (A), cell motility (B), and invasiveness (C) were confirmed using qRT-PCR, transwell migration (E, scale bar 100 μm), and Matrigel transwell invasion assay (F, scale bar 100 μm), respectively. qRT-PCR results were normalized to GAPDH. Data are presented as the mean ± standard deviation (SD) of triplicate experiments. *P < 0.05, **P < 0.01, ***P < 0.001 were analyzed using One-Way ANOVA for A, C, and D.

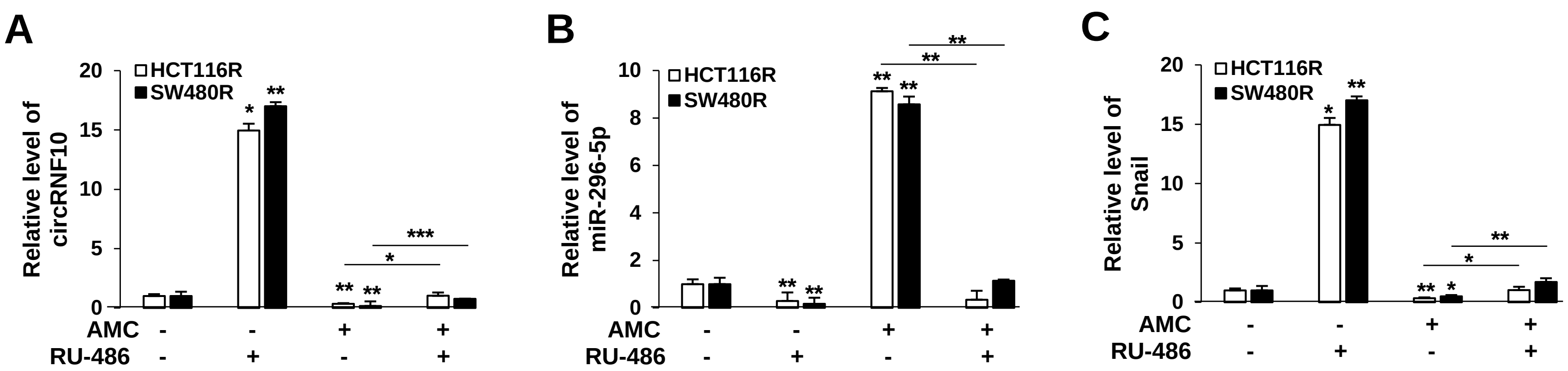

Supplementary Figure 7 | AMC inhibits tumorigenicity through the circRNF10/miR-296-5p/Snail axis. (A-C) After treating HCT116R and SW480R cells with AMC (80 μM) and the GR antagonist RU-486 (10 μM), mRNA levels of circRNF10 (A), miR-296-5p (B), and Snail (C) were determined by qRT-PCR. qRT-PCR results were normalized to GAPDH and U6. Data are presented as the mean ± standard deviation (SD) of triplicate experiments. *P < 0.05, **P < 0.01, ***P < 0.001 were based on One-Way ANOVA (A, B, and C).

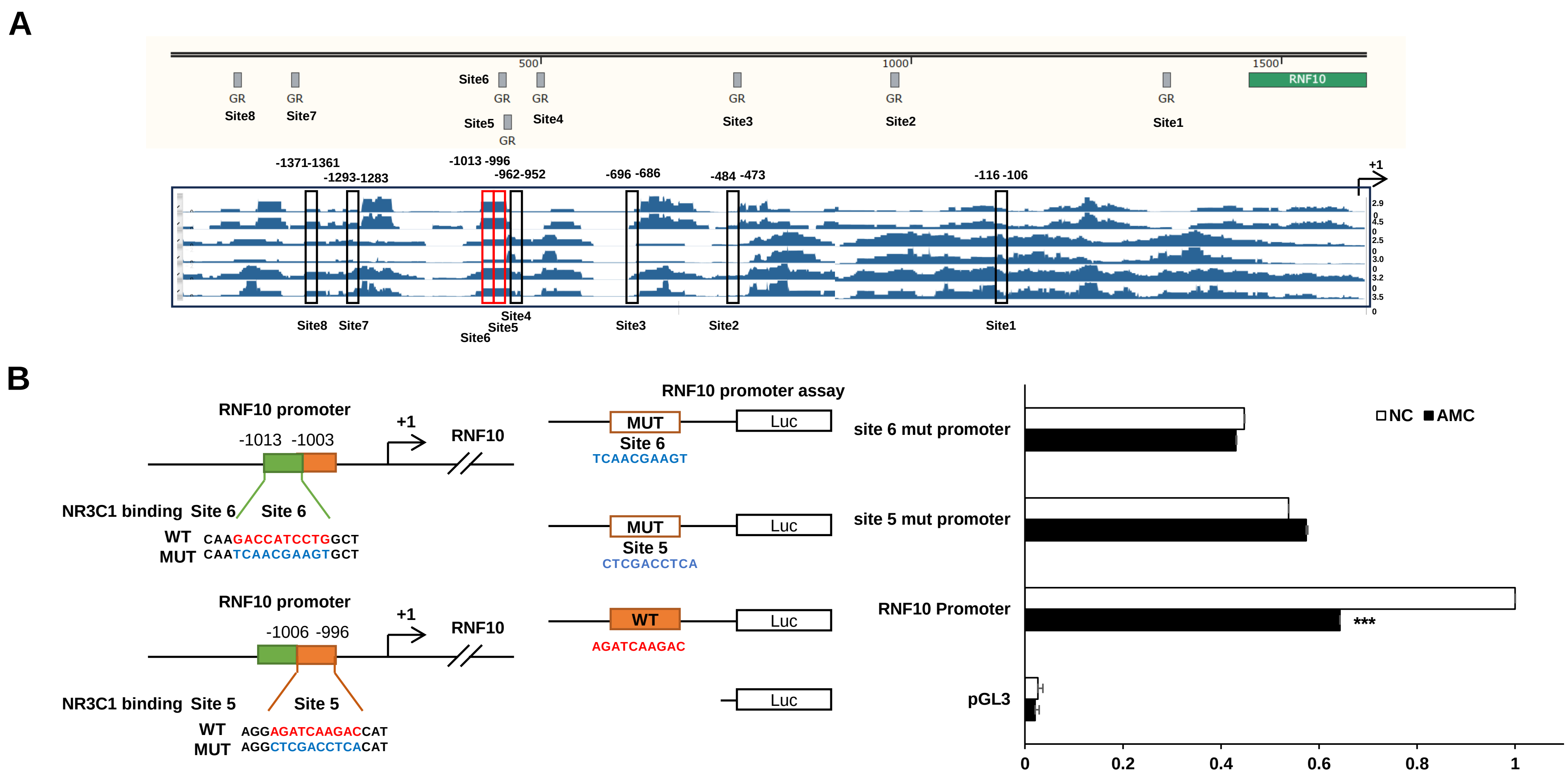

Supplementary Figure 8 | The glucocorticoid receptor inhibits its activation by directly binding to the circRNF10 promoter. (A) Top, a schematic diagram showing eight potential NR3C1 binding sites within the 1.5kb upstream promoter region of RNF10, predicted by AliBaba 2.1. Bottom, showing binding signals from ENCODE ChIP-seq data obtained from CRISPR-induced NR3C1 knockout HepG2 cell lines (GEO: GSE170210), indicating strong binding enrichment of NR3C1 at predicted sites 5 and 6. (B) Luciferase reporter analysis was performed on HCT116 cells transformed with wild-type (WT) or site-specific mutant (MUT) RNF10 promoter constructs after treatment with AMC (80 μM). Relative luciferase activity was calculated by normalizing firefly luciferase activity to renilla luciferase activity. Data are presented as the mean ± standard deviation (SD) of triplicate experiments. *P < 0.05, **P < 0.01, and ***P < 0.001 were based on Student's t-Test (B)

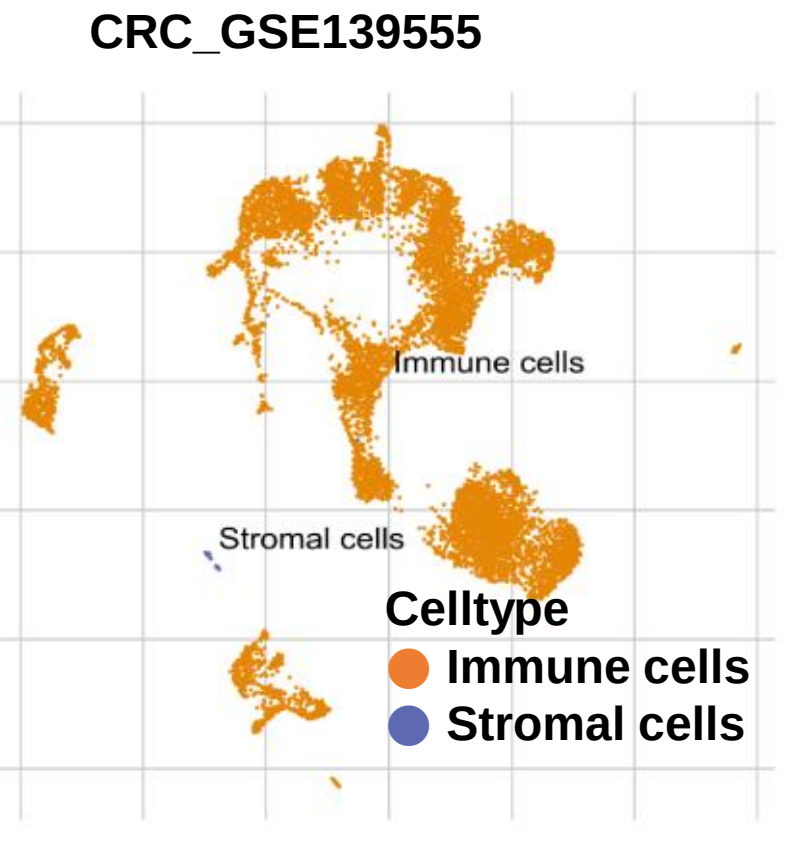

Supplementary Figure 9 | Single-cell RNA sequencing analysis of CRC revealed that Snail expression is associated with immune cells. The distribution of immune cells and stromal cells was confirmed through single-cell RNA sequencing analysis of the CRC dataset (CRC_GSE139555).

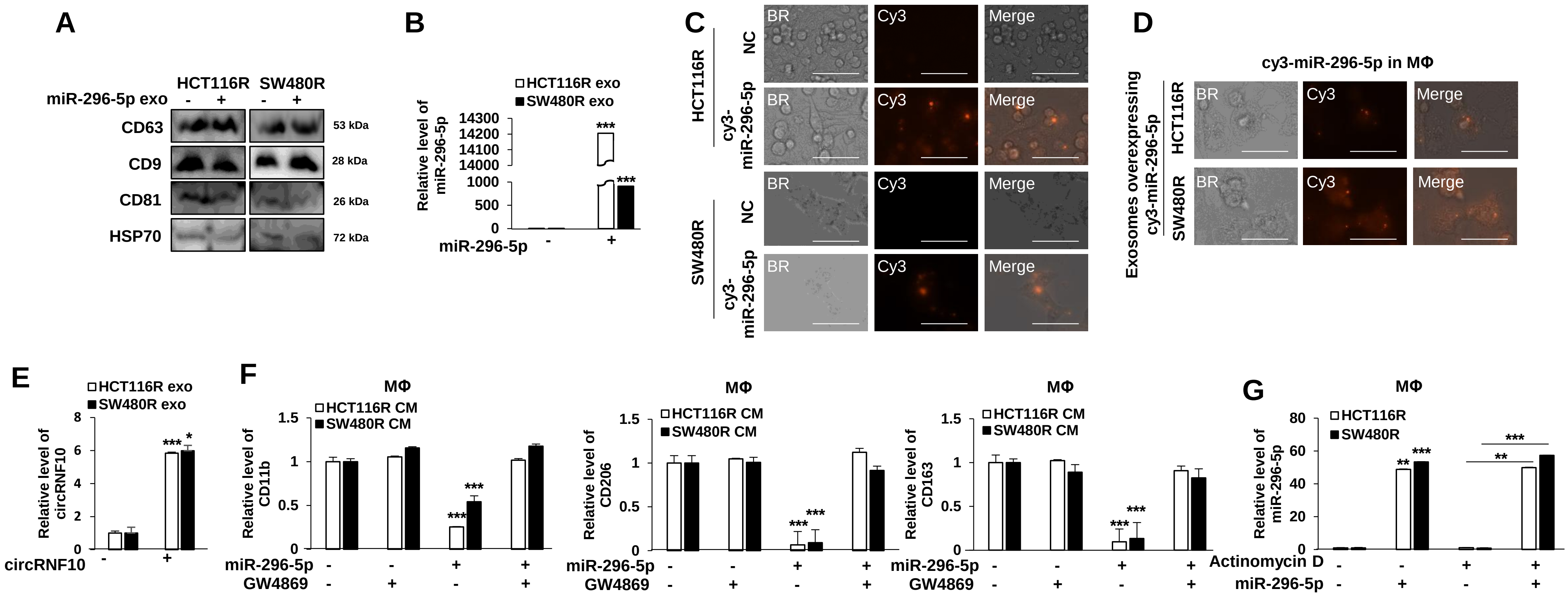

Supplementary Figure 10 | miR-296-5p is transferred from radioresistant CRC cells to macrophages via exosomes. (A-C) After transforming HCT116R and SW480R cells with Cy3-tagged miR-296-5p mimic, exosomes were isolated from the cells. The exosome markers (CD63, CD9, and CD81) (A), miR-296-5-p (B), and the fluorescence levels of Cy3 (C, scale bar 60 μm) were confirmed by western blot analysis, qRT-PCR, and fluorescence microscopy, respectively. qRT-PCR results were normalized to U6. (D) Cy3 was confirmed by fluorescence microscopy after treating macrophages with CM of HCT116 and SW480 cells transfected with cy3-miR-296-5p (B, scale bar 60 μm). (E) After overexpressing circRNF10 in HCT116R and SW480R cells, the level of circRNF10 in exosomes was confirmed by qRT-PCR. (F) miR-296-5p overexpressing HCT116R and SW480R cells were treated with CM containing exosome secretion inhibitor GW4869, and the abundance of M2 markers was confirmed using qRT-PCR. qRT-PCR results were normalized to GAPDH. (F) The levels of miR-296-5p were confirmed in macrophages after treating the CM of HCT116R and SW480R cells transfected with miR-296-5p mimic with Actinomycin D. qRT-PCR results were normalized to GAPDH and U6. Data are presented as the mean ± standard deviation (SD) of triplicate experiments. *P < 0.05, **P < 0.01, ***P < 0.001 were analyzed using Student's t-Test (B and D) or One-Way ANOVA (E and F), respectively.
