# Supplementary material for: Amcinonide Exhibits Antitumor Effects in Radioresistant Colorectal Cancer Through the circRNF10/miR‐296‐5p/Snail Axis
Source: Adv Sci (Weinh). 2026 Oct 26:e78502. Online ahead of print. doi: 10.1002/advs.78502 (PMC13710703; doi:10.1002/advs.78502)
Supplement: Supplementary file 2 [file ADVS-9999-e78502-s002.docx]

**Supplementary Table 1.** Primer sequences used in this study.

| Primers for qRT-PCR | Sequences (5'-3') | |
| --- | --- | --- |
| Snail | Forward | GTTTACCTTCCAGCAGCCCT |
|  | Reverse | GAGCCTTTCCCACTGTCCTC |
| Zeb1 | Forward | ATGACTGCCCTAACCACAGG |
|  | Reverse | TGCAGTCATCTCCACTCCAG |
| Twist | Forward | GAAGATCATCCCCACGCTG |
|  | Reverse | AGGAAGTCGATGTACCTGGC |
| Slug | Forward | ACAGCGAACTGGACACACAT |
|  | Reverse | TCACTCGCCCCAAAGATGAG |
| Vimentin | Forward | GCTTGGAAACATCCACATCG |
|  | Reverse | GAGAGGAAGCCGAAAACACC |
| GAPDH | Forward | CATCTCTGCCCCCTCTGCTGA |
|  | Reverse | GGATGACCTTGCCCACAGCCT |
| miR-296-5p | Forward | AGGGCCCCCCCTCAATCCTGT |
| miR-199a-5p | Forward | CCCAGTGTTCAGACTACCTGTTC |
| circRNF10 | Forward | CCAGTCAGGGCAGTCCCTC |
|  | Forward | GGCTTTGCAACCTCCTGTGG |
| RNF10 | Forward | GATGTTCGACAGCGTCACAGATATCTC |
|  | Reverse | CCCTTCCCTTCCTTTGCCCAG |
| TGF-β | Forward | GTGGAAACCCACAACGAAAT |
|  | Reverse | CGGAGCTCTGATGTGTTGAA |
| CD11b | Forward | ACGTAAATGGGGACAAGCTG |
|  | Reverse | GATCCTGAGGTTCCGTGAAA |
| CD206 | Forward | ACGGACTGGGTTGCTATCAC |
|  | Reverse | TGATCCCCAAAAGTGTGTCA |
| CD163 | Forward | CCAGTCCCAAACACTGTCCT |
|  | Reverse | CACTCTCTATGCAGGCCACA |
| Primers for overexpression vector | | **Sequences (5'-3')** |
| circRNF10 overexpression | Forward | GCGAATTCGATGTTCGACAGCGTCACAGATATCTC |
|  | Reverse | CGGCGGCCGCCTGGGCAAAGGAAGGGAAGGG |
| Snail overexpression | Forward | CGGGATCCATGCCGCGCTCTTTCCTC |
|  | Reverse | CCCAAGCTTTCCTCCCAGGCCCACCTG |
